# Capturing the antimicrobial profile of *Paeonia officinalis, Jasminum officinale* and *Rosa damascene* against methicillin resistant *Staphylococcus aureus* with metabolomics analysis and network pharmacology

**DOI:** 10.1038/s41598-024-62369-5

**Published:** 2024-06-13

**Authors:** Nourhan Hisham Shady, Fatma Alzahraa Mokhtar, Basma Khalaf Mahmoud, Ramadan Yahia, Ayman M. Ibrahim, Nada Ahmed Sayed, Mamdouh Nabil Samy, Mubarak A. Alzubaidi, Usama Ramadan Abdelmohsen

**Affiliations:** 1 Department of Pharmacognosy, Faculty of Pharmacy, Deraya University, Universities Zone, New Minia City, 61111 Egypt; 2 Center for Research and Sustainability, Deraya University, Universities Zone, New Minia City, 61111 Egypt; 3 Fujairah Research Centre, Sakamkam Road, Fujairah, United Arab Emirates; 4 Department of Pharmacognosy, Faculty of Pharmacy, Minia University, Minia, 61519 Egypt; 5 Department of Microbiology and Immunology, Faculty of Pharmacy, Deraya University, New Minia City, Minia Egypt; 6 Department of Pharmaceutical Chemistry, Faculty of Pharmacy, Deraya University, New Minia City, 61111 Egypt; 7 Faculty of Pharmacy, Deraya University, Universities Zone, New Minia City, 61111 Egypt; 8 Department of Biological Sciences, Faculty of Science, King Abdulaziz University, 21589 Jeddah, Saudi Arabia

**Keywords:** Peony, MRSA, Metabolomics, Network pharmacology, Microbiology, Computational chemistry

## Abstract

In the current study, we evaluated the in vitro antibacterial efficacy of the roots’ extracts of *Jasminum officinale*, *Rosa damascene* and *Paeonia officinalis* against MRSA (methicillin-resistant *Staphylococcus aureus*) by well diffusion technique. The root extract of *P. officinalis* exerted a potent anti-MRSA with MIC 0.4673 µg/ml, while both *J. officinale* and *R. damascene* exhibited very weak activity. Therefore, chemical profiling of the crude extract *P. officinalis* roots assisted by LC-HR-ESI-MS was performed and led to the dereplication of twenty metabolites of different classes, in which terpenes are the most abundant compounds. On a molecular level, network pharmacology was used to determine the targets of active metabolites to bacterial infections, particularly MRSA. Online databases PubChem, UniProt, STRING, and Swiss Target Prediction were used. In addition to using CYTOSCAPE software to display and analyze the findings, ShinyGO and FunRich tools were used to identify the gene enrichment analysis to the set of recognized genes. The results detected the identified metabolites were annotated by 254 targets. ALB, ACHE, TYMS, PRKCD, PLG, MMP9, MMP2, ERN1, EDNRA, BRD4 were found to be associated with MRSA infection. The top KEGG pathway was the vascular smooth muscle contraction pathway according to enrichment FDR. The present study suggested a possible implication of *P. officinalis* roots as a potent candidate having a powerful antibacterial activity against MRSA.

## Introduction

Methicillin-resistant *Staphylococcus aureus* (MRSA) is one of the major causes of hospital- and community-associated infections^1^. MRSA infections have the capability to resist the impacts of many common antibiotics such as methicillin, penicillin and other common antibiotics^2^. MRSA infections are more complicated to be treated. Moreover, can influence the bloodstream, heart, bones, lungs, and joints of MRSA casualties, and can transmit from an object which contains MRSA to a human, or from a human carrier to another human^3^. *Staphylococcus aureus* can cause various infections, ranging from common skin and respiratory problems to life-threatening conditions like necrotizing fasciitis and a severe form of *pneumonia* (necrotizing pneumonia)^1^. MRSA can cause different types of infections like Healthcare associated MRSA, Community associated MRSA and Livestock associated MRSA infections^4^. Natural products exerts a great role in MRSA prevention, the herbals extracts of *Garcinia mangostana* and *Quercus infectoria* have considerable activity against MRSA^5^. Furthermore, curcumin, garlic, ginger, Thai longan honey, Juncus and Luzula species, Greek oregano, Baru plant, and Lichen are natural products that showed great potential against drug-resistant *S. aureus*^3^. The usage of natural products in therapeutic management against MRSA caused diseases have a benefit over the application of synthetic drugs due to the decreased side effects of natural products extracts. In traditional Chinese medicine *Paeonia* genus is considered the only genus in the family *Paeoniaceae* that holds a great potential for use in medicine. Paeonia genus include 33 known species, however there are a different opinions on the number of species that can be distinguished ranging from twenty five to forty^6^. Peony is widely planted and distributed in warm regions of Europe and Asia, where one of the natural spreading centers of wild peony species is China^7^. Furthermore, Paeonia roots has a variety of medicinal applications such anti-inflammatory and antipyretic agents as well as in treatment of critical disorders such as cardiovascular and female genital disorder^8^. The roots of *Paeonia officinalis* are rich with alkaloids, tannins, saponins, glycosides, carbohydrates, flavonoids, terpenes, steroids and proteins^9,10^. Additionally, the roots contain asparagin, benzoic acid, flavonoids, paeoniflorin, paeonin, paeonol, protoanemonin, tannic acid, triterpenoids, and volatile oil^8^. *P. officinalis* is considered to be a dietary supplement that exhibited antimicrobial and antimalarial activities with no apparent cytotoxicity against mammalian cells^8^. Herein, we aimed to examine the in vitro antibacterial efficacy of the crude extracts of *J. officinale*, *R. damascene* and *P. officinalis* roots against MRSA. Furthermore, metabolomics profiling of the crude extract was performed to highlight the bioactive compounds involved in this activity. As well as, on a molecular level, network pharmacology was used to determine the targets of active metabolites to bacterial infection.

## Materials and methods

### Plant collection

The *P. officinalis* roots, *J. officinale* and *R. damascene* were collected from public nurseries in January 2021, Minia governorate, Egypt, where the permissions were obtained from an appropriate governing body to a piece of legislation that permits this. The plant authentication was identified by Prof. Dr. Nasser Barakat (Department of Botany, Faculty of Science, Minia University, Minia, Egypt) comply with the IUCN Policy Statement on Research Involving Species at Risk of Extinction and the Convention on the Trade in Endangered Species of Wild Fauna and Flora. A voucher specimen of The *P. officinalis* roots' taken number (Mn-ph-Cog-062) has been deposited in the Herbarium of Pharmacognosy Department, Faculty of Pharmacy, Minia University, Minia, Egypt. While A voucher specimen *of J. officinale, R. damascene* taken numbers (Du-Ph-Cog-10), (Du-Ph-Cog-11), respectively have been deposited in the Herbarium of Pharmacognosy Department, Faculty of Pharmacy, Deraya University, New Minia, Egypt.

### Extraction of *P. officinalis*, *J. officinale* and *R. damascene* Roots

The dried roots of each *P. officinalis*, *J. officinale* and *R. damascene* (650 g of each) were macerated separately in 95% methanol at room temperature until exhausted. After that, reduced pressure was used to get rid of the alcohol, affording a viscous syrupy residues (60, 72, 65 g) for *P. officinalis*, *J. officinale* and *R. damascene* consequently.

### Metabolomics analysis

High resolution-Liquid chromatography-Mass spectrometry (HR-LC-MS) was carried out using a Synapt G2 HDMS quadrupole time-of-flight hybrid mass spectrometer (Waters, Milford, CT, USA). The sample (2 µL) was injected into the BEH C18 column, adjusted to 40 ^◦^C, and connected to a guard column. A gradient elution of mobile phase was used, starting from 100% water in 0.1% formic acid as solvent A to 100% acetonitrile in 0.1% formic acid as solvent B. MZmine 2.12 (San Diego, CA, USA) was employed for differential investigation of MS data, followed by converting the raw data into positive and negative files in mzML format with ProteoWizard (Palo Alto, CA, USA). The detected compounds were finally annotated by comparison with the Dictionary of Natural Products (DNP) (Dictionary of Natural Products 2020) and METLIN (METLIN 2020) databases^11–13^.

### Evaluation of antimicrobial activity using the well-diffusion assays technique

The antimicrobial activity of The *P. officinalis*, *J. officinale* and *R. damascene* roots were evaluated against pathogenic bacteria *Methicillin resistant Staphylococcus aureus* (ATCC 33591) through Well-diffusion assays technique. Whereas, the dried extracts were dissolved in dimethyl sulfoxide (DMSO). The agar plates were prepared as follows: a pure culture of the MRSA strains was grown in nutrient broth at 37 °C for 18–24 h in shaker incubator until the final concentration was 108 CFU/ml (the final concentration was adjusted by sterilized normal saline). Each twenty ml plain nutrient agar was poured to a sterile petri dish. A six mm well was punched in the solid agar plates via a sterile cork-borer. Each plate was surface inoculated by 100 μl broth culture of the tested strain in triplicates. Eight serial dilutions of each extract were made (% w/v) in dimethylsulfoxide (DMSO) (10% aqueous) solvent as follows: 200, 100, 50, 25, 12.5, 6.25, 3.12 and 1.56 μg/ml and sterilized by filtration by passing through 0.22 μm membrane filter. Plain DMSO was used as a control. 50 μl of each tested dilution was pipetted to the wells of the inoculated agar plates aseptically. The plates were incubated at 35 °C for 24 h. After incubation, the inhibitory zones were measured in millimeters and the minimum inhibitory concentration was calculated. Ciprofloxacin was used as a positive control.

## Computational pharmacology study

### Networks construction

#### Plant-metabolite network

Based on chemical analysis of *P. officinalis* roots using** (**HR-LC-MS), metabolic profiling was identified, the dereplicated 20 metabolites were connected to the plant in a simple network.

#### The metabolites–targets network

The targets annotated by the identified metabolites were predicted using data from PubChem^14^, BindingDB^15^ and SawissTargetPrediction^16^ databases, the top targets were annotated based on similarity index of more than 0.7 in BindingDB and choose the top targets in Swiss Target Prediction database^16^ using canonical smiles for each structure as input data method, the human species (*Homo sapiens*) was selected.

#### Targets–infections network

DisGenet^17^ online database was used to identify the targets for certain infections, and particular filter terms; ‘infection’, ‘MRSA infection’ then we used filter option in downloaded DisGenet output to refined the results.

#### Protein–protein interaction

The protein–protein interaction was anticipated through the STRING online database (https://string-db.org/cgi/network?taskId=bIDN4htc9NBY&sessionId=bZWvNlZHMn9h)^18^ as trustworthy data sources for speculating on the interactions between proteins. As a starting point, we used *Homo sapiens* as the species and a confidence threshold of 0.4 to choose the target proteins.

#### Complete pharmacology network

Combining the previous networks in a single network result in the complete network (The network of the plant–metabolites–targets–infections).

#### Networks construction

All the formed networks were visualized, analysed and illustrated using the Cytoscape software version 3.9.0.

#### Gene ontology and enrichment analysis

To identify the cellular components, molecular functions, and biological processes that were influenced by this set of genes, gene ontology and enrichment analysis were carried out on all targets of the active metabolites, we used FunRich version 3.1.3^19^, the enrichment analysis was done using KEGG database^20^ and ShinyGo database (a graphical gene set enrichment tool)^21^.

#### Molecular docking

Molecular docking was conducted utilizing the AutoDock software. The crystal structures of the proteins were obtained from the RCSB Protein Data Bank (http://www.rcsb.org/). To prepare the input files for molecular docking, Discovery Studio (DS) 2016 client and AutoDock tools bundled with MGL tools (version 1.5.7) were employed. The proteins were prepared by eliminating water molecules and small molecular ligands, and the addition of polar hydrogens and charges. The three-dimensional structures of the ligands were retrieved from the PubChem database as a single file in 3D-spatial data file (SDF) format. The ligand structures were imported into DS 2016, minimized using a universal force field, and saved in PDB format. Gasteiger charges and polar hydrogens were incorporated, and the ligands were configured for the rotatable bond. Subsequently, the prepared protein and ligand files were converted into PDBQT format, which served as the input for AutoDock 1.5.7 for molecular docking. The active site of the ligands was determined based on a literature survey and selected as the active grid center. The dimensions of the grid box were adjusted to encompass all atoms of the ligands. The molecular docking was then performed, and the protein–ligand conformation with the lowest binding energy was selected and visualized using proteins plus server (https://proteins.plus/).

## Results

### The antimicrobial potential of *P. officinalis* root extract against MRSA

The antimicrobial activity examination of the crude extracts of three roots; *J. officinale*, *R. damascene* and *P. officinalis* roots against MRSA were performed via the well-diffusion assays revealing the highest antibacterial potency of *P. officinalis* extract against MRSA with MIC 0.4673 µg/ml, while the other two extracts showed no activity (> 100 µg/ml). The MIC for ciprofloxacin against MRSA was 19.2 µg/ml. Peony plants and their metabolites are well known with their varied biological activities including; anti-inflammatory, immunomodulatory, neuroprotective, antiviral, antidiabetic, etc.^22^. HR-LC-ESI-MS analysis of the crude *P. officinalis* extract was performed for highlighting the metabolites responsible for the strong anti-MRSA potency.

### Quadrupole time-of-flight mass spectrometry (QTOF-MS) assisted dereplication of the chemical constituents in *Paeonia officinalis* root extract.

**Figure 1:**
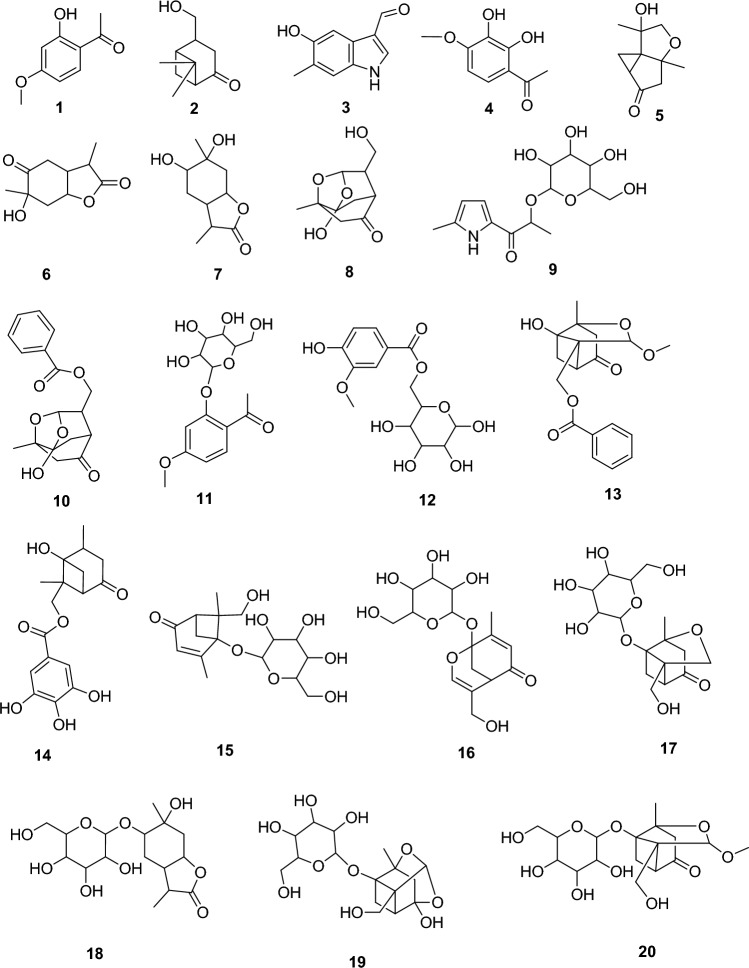
Chemical structures of the dereplicated secondary metabolites (1–20) from *P. officinalis* root.

Metabolomics profiling assisted by HR-LC-ESI-MS analysis of the crude *P. officinalis* extract (Fig. 1) led to the identification of a wide range of phytoconstituents, whereas terpenoids and phenolics are the prominent classes such as Paeonol **(1)**^23^, Paeonilactinone (**2**)^24^, 5-Hydroxy-6-methyl-1H-indole-3-carboxaldehyde (**3**)^25^, 2′,3′-Dihydroxy-4′-methoxyacetophenone (**4**)^26^, Paeonisothujone (**5**)^27^, Paeonilactone A (**6**)^28^, Lactinolide (**7**)^24^, Paeonisuffral (**8**)^29^, Paesuffrioside **(9)**^30^, Paeoniflorigenone (**10**)^31^, Paeonosid (**11**)^32^, Mudanoside A **(12)**^33^, Paeonidangenin **(13)**^34^, Paeoniflorone **(14)**^34^, Mudanpioside F (**15**)^35^, Paeonin B (**16**)^36^, 1-O-β-d-Glucopyranosylpaeonisuffrone (**17**)^37^, 1,6-Dihydroxy-p-menthan-9,3-olide 6-O-β-d-Glucopyranoside (**18**)^38^, 8-Debenzoylpaeoniflorin (**19)**^39^, Debenzoylpaeonidanin (**20**)^29^. Literature survey of these metabolites revealed varied bioactivities, whereas Paeoniflorigenone (**10**) is one of the dereplicated monoterpenes was reported to have notable in vitro antibacterial potency through strong inhibition of the DNA polymerase activity on multiple nucleotide addition assays method^40^.

### Metabolites–targets network

**Figure 2:**
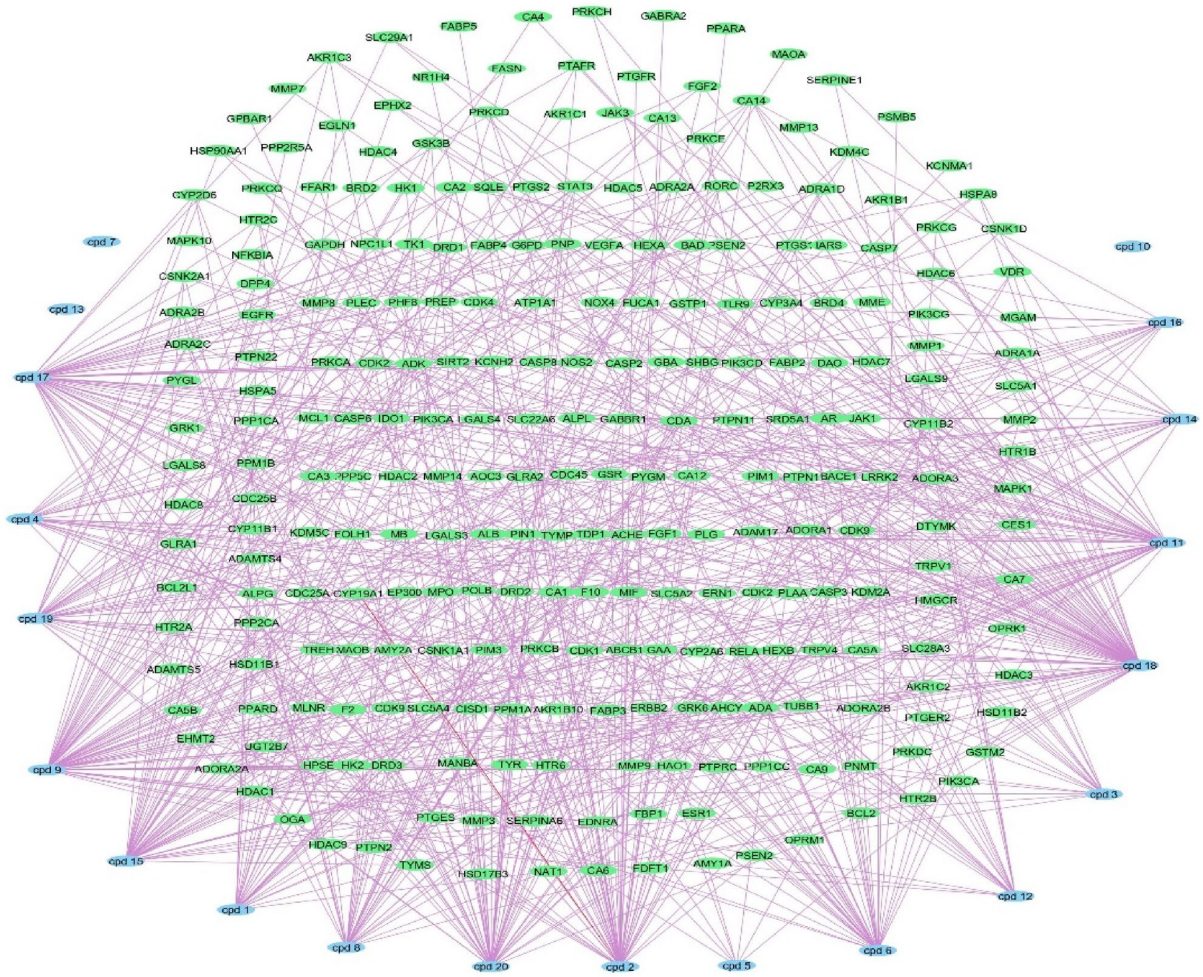
Metabolites–targets network: a network of annotated targets related to the identified metabolites from the *P. officinalis*, blue rectangles represent identified metabolites, green rectangles represent targets.

254 target genes were found to be targeted by the identified metabolites the *p. officinalis*, a network (metabolites–targets) was formed, the network composed of 285 nodes and 683 edges, with network centralization 0.289, no targets were found for compounds 7, 10, 13 (Fig. 2 and Table S1) the formed network identified CDA, ADA, ADK and ADORA2A genes as the top targets related to the metabolites with 9 edges for each.

### Targets–infections network

**Figure 3:**
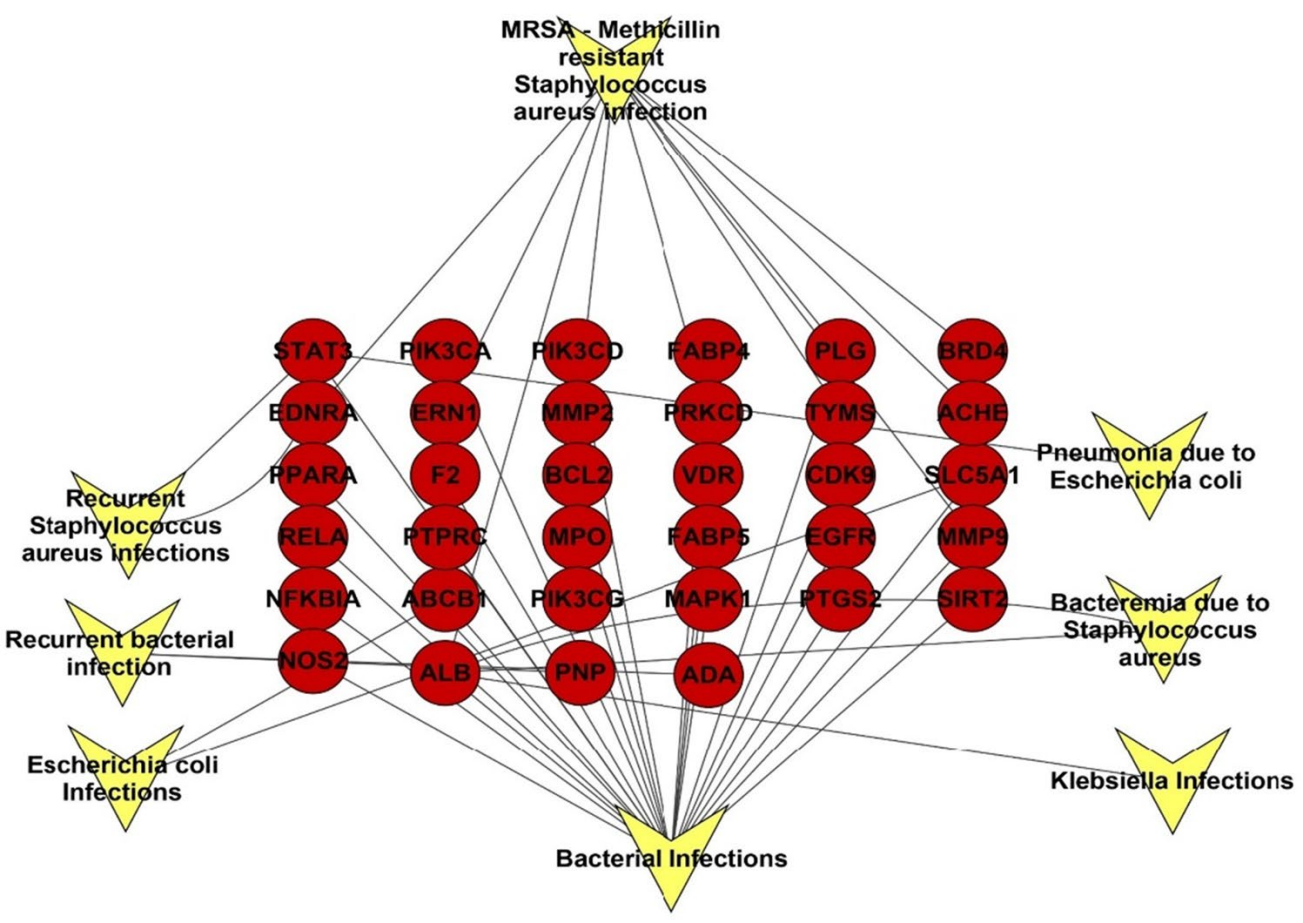
Targets–infections network, a network describing the association between the identified targets and different types of infections, red circles represent targets, inverted arrow heads represent types of infections.

A DisGeNET database results of the targets revealed an association of the targets to bacterial infectious diseases especially MRSA infections, the analysis of the formed network of gene–infection association revealed the abundance of 42 nodes representing types of bacterial infections and related targets among our targets set, and 58 edges with characteristic path length of 2.496 and network centralization of 0.635. Focusing on MRSA infection, the targets namely ALB, ACHE, TYMS, PRKCD, PLG, MMP9, MMP2, ERN1, EDNRA, BRD4 were found to be the genes affecting the MRSA infection (Fig. 3).

### Protein–protein interactions

**Figure 4:**
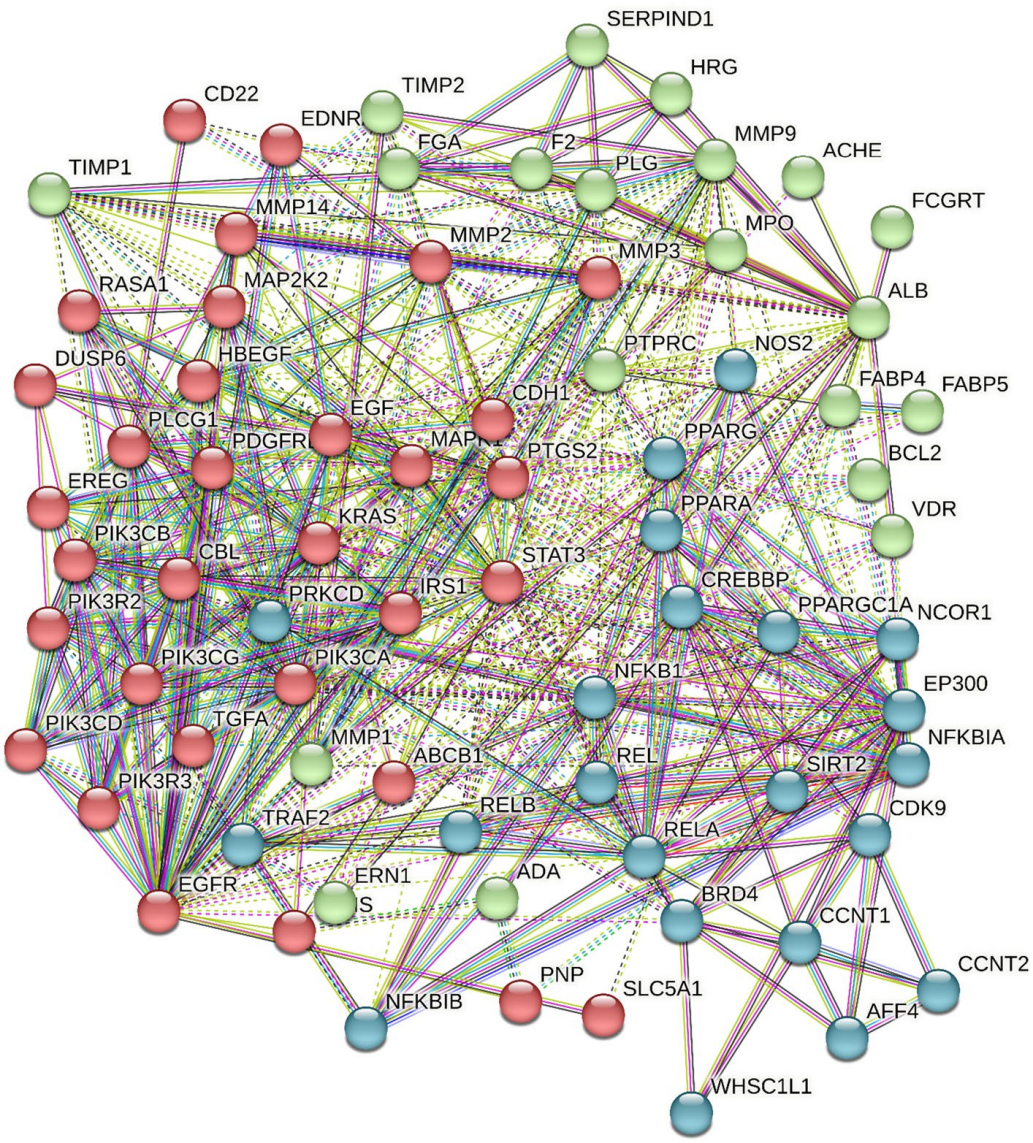
Protein protein interaction, interaction between target proteins targeted by identified metabolites from *P. officinalis* the interaction is shown in 3 clusters.

All the targets were detected to determine the interactions between individual proteins and clusters, the formed protein–protein interaction network composed of 74 nodes, and 590 edges with average node degree of 15.9 and average local clustering coefficient equal 0.654 (Fig. 4).

### Complete pharmacology network

**Figure 5:**
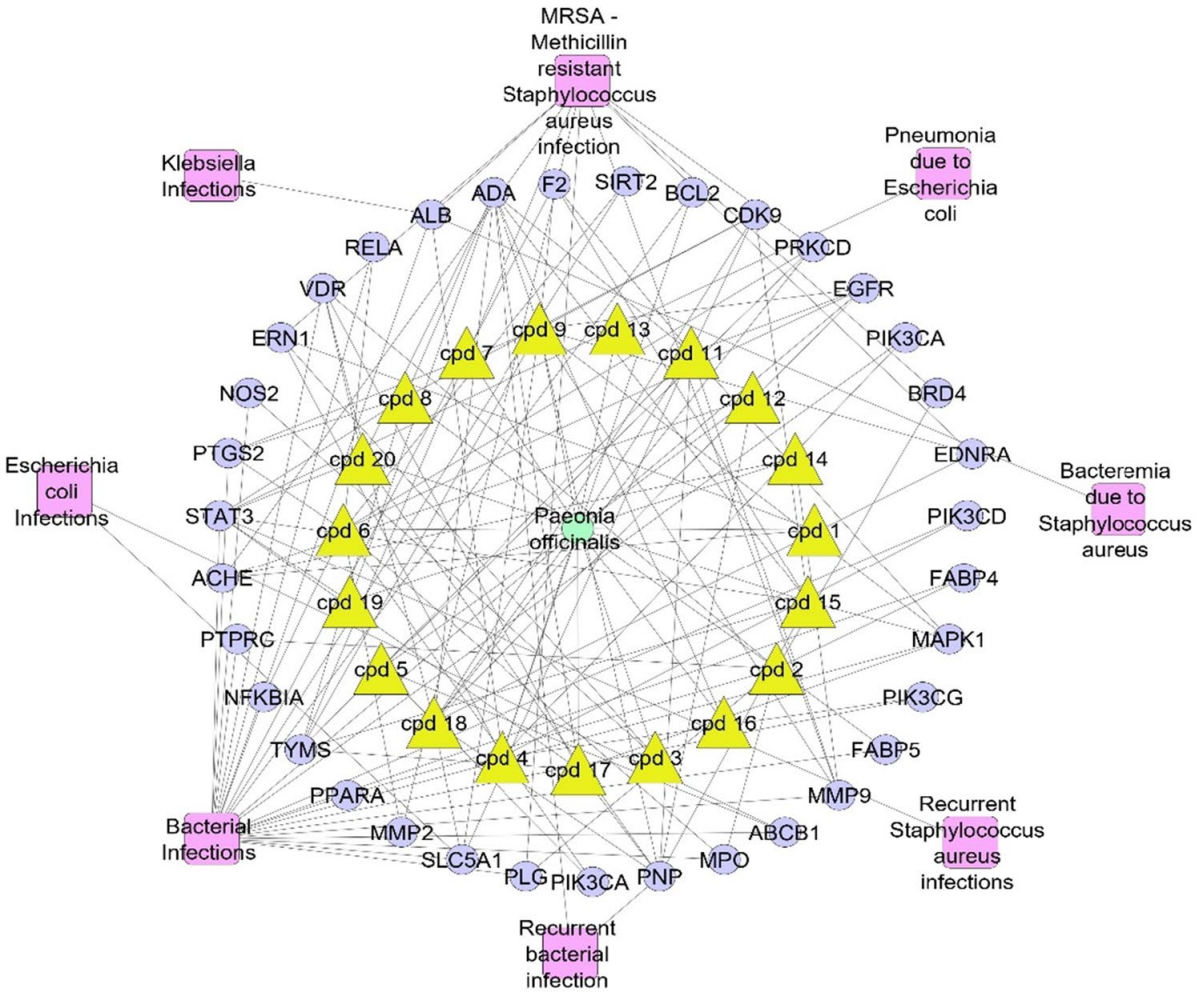
Complete pharmacology network; (plant–metabolite–targets–infection); green circle is the plant name, yellow triangles are the active identified metabolites, violet circles represent genes correlated to infection, pink rectangles represent different types of infection.

By merging the previous networking in a way that focus on the only genes associated to bacterial infections a complete network pharmacology was evolved that connected (plant–metabolites–targets–infection), in this network we neglected compounds that were not correlated to target genes as well as neglected the targets that were not correlated to bacterial infection (Fig. 5).

### Gene ontology and enrichment analysis

**Figure 6:**
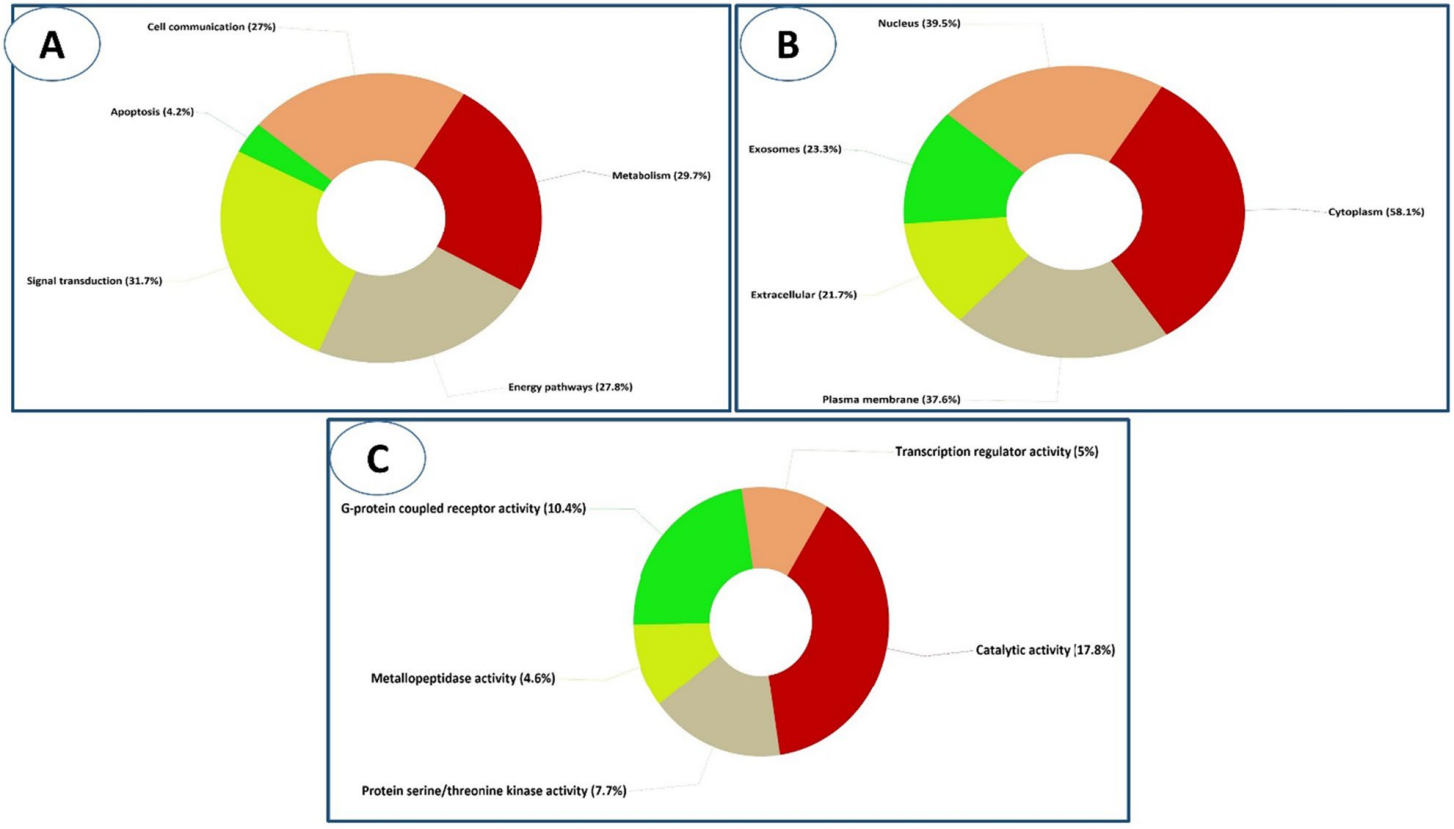
Gene enrichment analysis of target genes of *P. officinalis* active metabolites showing (**A**) biological processes, (**B**) cellular components, and (**C**) molecular functions.

**Figure 7:**
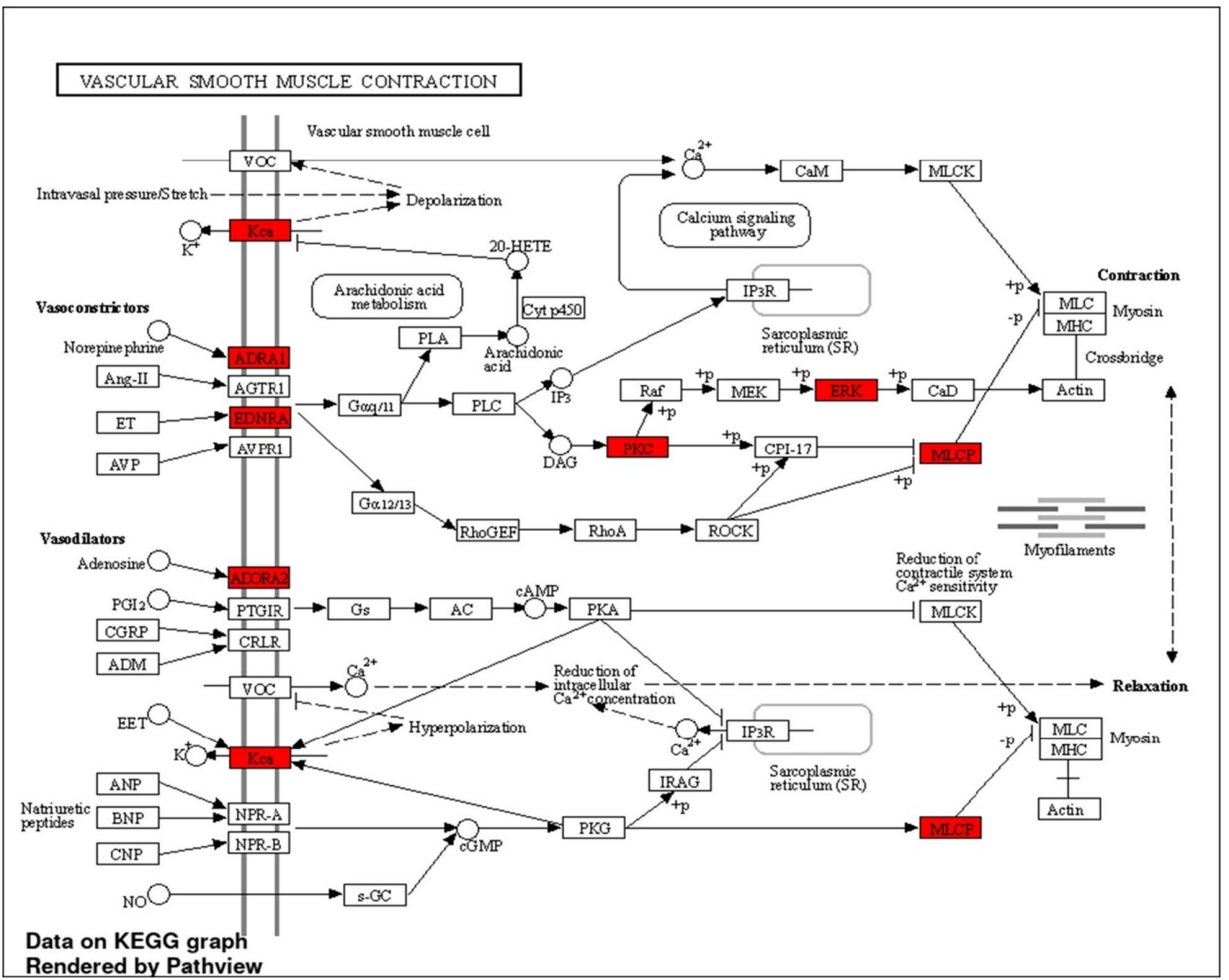
KEGG illustrating diagram of biological pathways showing the vascular smooth muscle contraction as the top pathway and evolved genes.

We utilised FunRich software to perform a gene ontology and enrichment analysis on all the active metabolites' targets to determine the cellular components, molecular functions, and biological processes that are affected by this group of genes. The results confirmed that signal transduction, metabolism and the energy pathway are the most prominent biological processes, in that order (Fig. 6A). The Cytoplasm, the Nucleus and the Plasma membrane are the top three cellular component (Fig. 6B). Catalytic activity was the top molecular function, followed by G-protein coupled receptor activity and protein serine/threonine kinase activity (Fig. 6C and Table S2). Using an enrichment analysis implemented in ShinyGO v0.741, we were able to identify the most significant biological pathways associated with the target genes; these pathways include the vascular smooth muscle contraction pathway (Fig. 7) was the top KEGG. pathway according to enrichment FDR (Fold Discovery Rate) followed by proteoglycans in cancer and calcium signaling pathway (Table S3).

### Molecular docking studies

Infections can be difficult to treat and can lead to serious complications, including sepsis and death. The genes mentioned “ALB, ACHE, TYMS, PRKCD, PLG, MMP9, MMP2, ERN1, EDNRA, BRD4” are related to MRSA as they contribute to the development and spread of MRSA. For example, ALB, PLG, MMP9, and MMP2 have been implicated in the host immune response to bacterial infections, including MRSA. ALB, which codes for the protein albumin, is involved in the transport of various substances throughout the body, including drugs and toxins. For instance, albumin has also been shown to have an antimicrobial effect, as it can bind to and neutralize bacterial toxins^41^. Inhibition of acetylcholinesterase (AChE) led to improved survival rates in mice infected with bacteria in a manner that was dependent on the dosage administered^42^. MMP9 and MMP2 are matrix metalloproteinases that are involved in tissue remodeling and are thought to play a role in the host response to bacterial infections^43^. In addition, ERN1 codes for the protein kinase RNA-like endoplasmic reticulum kinase, which is involved in the response to cellular stress. It has been shown to be important in the response to bacterial infections, as it can activate the immune response by promoting the production of pro- inflammatory cytokines. EDNRA codes for the endothelin receptor type A, which plays a role in the regulation of blood pressure and inflammation. It has been shown to be important in the response to bacterial infections, as it can activate the immune response by promoting the production of pro-inflammatory cytokines^44^.

The molecular docking approach was employed to investigate the binding affinities and potential interactions of the compounds with the target proteins. The twenty compounds were subjected to molecular docking analysis against possible potential target proteins encoded by the genes ACHE, TYMS, PRKCD, MMP9, MMP2, ERN1 and BRD4. The crystal structures of these targets were selected from the Protein Data Bank (PDB), including acetylcholine esterase (PDB ID: 4EY7), (PDB ID: 6QXG), protein kinase C delta (PDB ID: 1PTR), matrix metalloprotease 9 (MMP9) (PDB ID: 1GKC), MMP2 (PDB ID: 1HOV), inositol requiring enzyme 1 (IRE1) (PDB ID: 4U6R), and Bromodomain-containing protein 4 (PDB ID: 3MXF). The docking scores and binding affinities obtained from the docking are summarized in Table 1. The results presented in the table provide insights into the putative interactions and binding strengths of the compounds with the target proteins associated with MRSA-related processes.

**Table 1:** Molecular docking scores of twenty compounds against several biological targets.

Compound no.	Binding energy (kcal/mol)						
	4EY7	6QXG	1PTR	1GKC	1HOV	4U6R	3MXF
1	− 5.18	− 4.52	− 4.66	− 5.80	− 5.44	− 5.37	− 5.09
2	− 5.16	− 4.45	− 4.61	− 5.91	− 5.93	− 5.47	− 4.60
3	− 5.06	− 4.43	− 4.47	− 5.83	− 5.49	− 5.16	− 5.19
4	− 5.33	− 4.85	− 4.89	− 5.49	− 6.37	− 5.61	− 5.47
5	− 5.43	− 450	− 4.46	− 5.69	− 5.75	− 5.75	− 4.74
6	− 5.78	− 4.81	− 4.73	− 6.03	− 5.46	− 5.25	− 5.36
7	− 5.76	− 4.94	− 4.82	− 6.10	− 5.03	− 5.49	− 5.69
8	− 5.57	− 4.79	− 5.02	− 5.51	− 5.94	− 5.28	− 3.96
9	− 7.80	− 5.92	− 5.54	− 7.91	− 7.12	− 6.62	− 6.63
10	− 7.16	− 5.48	− 5.37	− 7.03	− 7.84	− 6.29	− 5.84
11	− 7.67	− 6.19	− 5.62	− 7.97	− 8.29	− 7.07	− 6.41
12	− 7.30	− 6.16	− 5.83	− 8.50	− 8.60	− 6.88	− 7.00
13	− 7.48	− 5.83	− 5.02	− 6.65	− 8.27	− 6.79	− 5.53
14	− 7.22	− 6.28	− 5.28	− 7.06	− 7.27	− 6.73	− 5.88
15	− 7.19	− 5.94	− 5.58	− 6.35	− 6.64	− 6.24	− 5.34
16	− 8.07	− 5.97	− 5.99	− 6.41	− 6.47	− 6.98	− 5.75
17	− 7.74	− 6.16	− 5.59	− 6.54	− 7.86	− 6.48	− 5.45
18	− 7.56	− 6.24	− 5.78	− 7.72	− 7.01	− 6.58	− 6.83
19	− 7.88	− 5.97	− 5.18	− 6.06	− 6.70	− 6.65	− 5.55
20	− 7.72	− 6.22	− 5.62	− 6.74	− 7.87	− 6.78	− 5.37
Co-crystallized ligand	− 8.73	− 6.97	− 6.70	− 7.92	− 15.19	− 11.55	− 7.2

A lower binding energy indicates a stronger binding affinity, which can be a desirable property for a drug molecule. In this analysis, the more negative value, the stronger binding affinity. All the selected compounds showed favorable binding, demonstrating ∆G (binding free energies) values in negative kcal mol^−1^.

**Figure 8:**
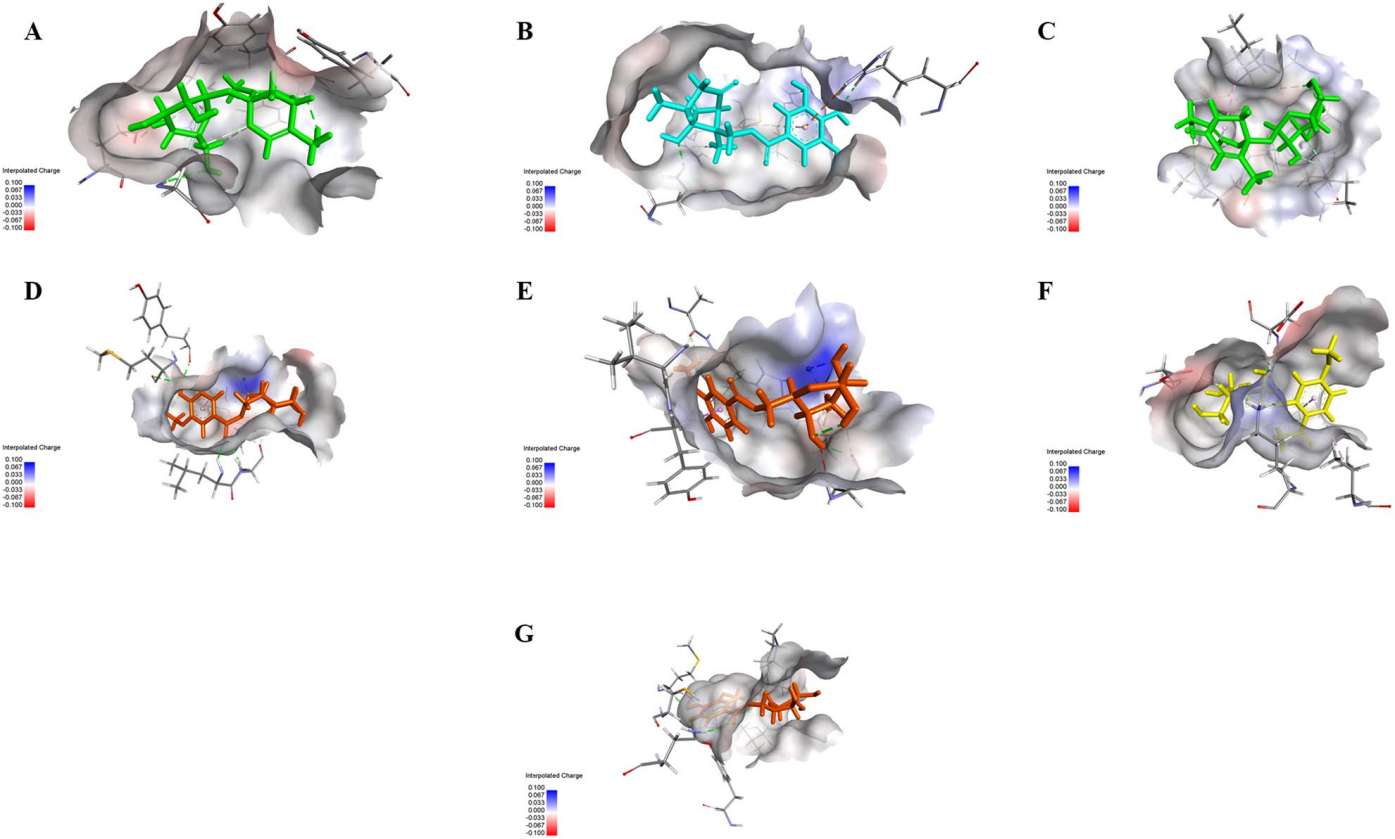
3D Binding mode of (**A**) Paeonin B 16 with acetylcholine esterase (PDB ID: 4EY7), (**B**) Paeoniflorone 14 with thymidylate synthase (PDB ID: 6QXG), (**C**) Paeonin B **16** with protein kinase C delta (PDB ID: PKCD), (**D**) Mudanoside A 12 with MMP9 (PDB ID: 1GKC), (**E**) Mudanoside A 12 with MMP2 (PDB ID: 1HOV), (**F**) Paeonosid **11** with inositol requiring enzyme 1 (IRE1α) (PDB ID: 4U6R), and (**G**) Mudanoside A **12** with Bromodomain-containing protein 4 (PDB ID: 3MXF)**.**

Upon examining the data, there is significant variability in the binding energies of the compounds across the different targets. This implies that these compounds exhibit varying affinities and specificities towards the biological targets. For instance, compounds **11** and **12** consistently display the lowest binding energies across most targets, suggesting a high affinity for these targets. Also, it can be inferred that compounds **11** and **12** possess the potential to act as multi-target agents (Fig. 8). Conversely, compounds **1**–**8** generally exhibit higher binding energies, indicating weaker interactions.

Furthermore, it is obvious that certain compounds demonstrate target-specific binding patterns. For instance, compounds **19, 10**, and **13** consistently display low binding energies for target 4EY7, and 1HOV, respectively, whereas they have relatively higher binding energies for other targets.

**Figure 9:**
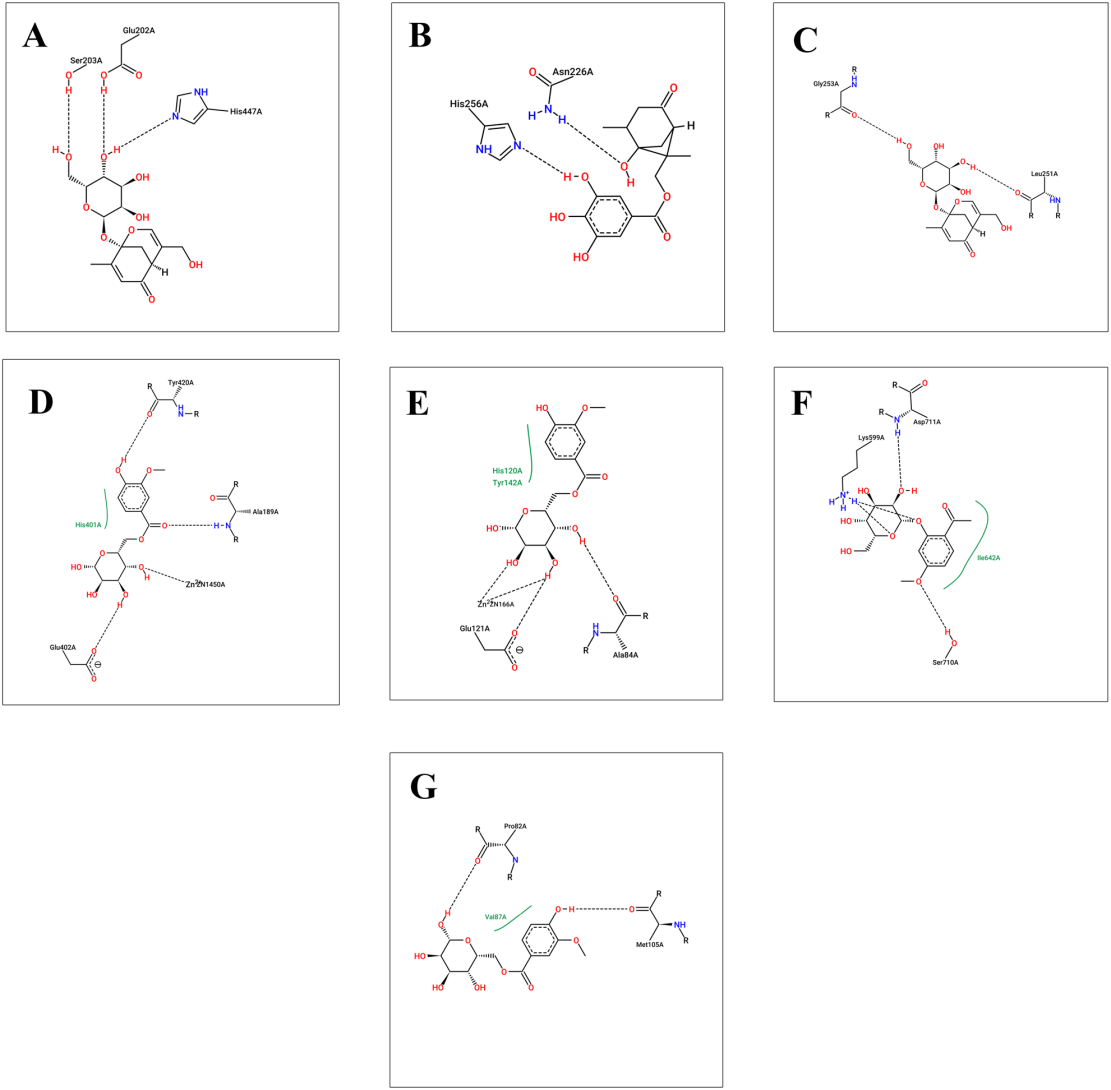
2D diagram for binding interactions (**A**) Paeonin B **16** with acetylcholine esterase (PDB ID: 4EY7), (**B**) Paeoniflorone **14** with thymidylate synthase (PDB ID: 6QXG), (**C**) Paeonin B **16** with protein kinase C delta (PDB ID: PKCD), (**D**) Mudanoside A **12** with MMP9 (PDB ID: 1GKC), (**E**) Mudanoside A **12** with MMP2 (PDB ID: 1HOV), (**F**) Paeonosid **11** with inositol requiring enzyme 1 (IRE1) (PDB ID: 4U6R), and (**G**) Mudanoside A **12** with Bromodomain-containing protein 4 (PDB ID: 3MXF).

Previous studies explained the importance of the catalytic triad residues Ser203, Glu334 and His447 in the AChE active site^45^. Docking studies revealed that Ser203, Glu202, and His447 amino acids stabilize the compound **16** in the active site of AchE (Fig. 9A). Moreover, compound **16** was able to bind significantly with protein kinase C by H-bonding with Gly253 and Leu251 (Fig. 9C). For thymidylate synthase (PDB ID: 6QXG), the co-crystallized ligand showed H-bonding interactions of ASP 218 with a C=O group and ASN 226 with N–H and a C=O group at the 3 and 4 positions. The binding pattern of compounds **14** was found to be similar to the thymidylate synthase protein. The compound forms hydrogen bond interactions, depicted as dotted lines in Fig. 9B, with Asn226 and His256. These interactions contribute to the stability and specificity of the compound's binding.

Matrix metalloproteinase 2 (MMP2) and matrix metalloproteinase 9 (MMP9) are both members of the matrix metalloproteinase family and share structural similarities. They both possess a similar overall domain organization, consisting of a prodomain, a catalytic domain, and a hemopexin-like domain. The catalytic domain is the core region responsible for the enzymatic activity of MMP2 and MMP9. It contains the zinc-binding motif and catalytic residues necessary for the cleavage of specific substrates. The catalytic domains of MMP2 and MMP9 share high sequence homology, indicating functional conservation^46^. These findings may explain the ability of compound **12** to act on MMP2 and MMP9 with the lowest binding energies among other compounds and co-crystallized ligand. Sugar moiety of compound **12** chelates with zinc ion in both MMP2 and MMP9 (Fig. 9D,E). In case of inositol-requiring enzyme 1 (IRE1), the key interaction points indicate several fundamental groups with favorable electrostatic interaction energy with the exogenous ligands, namely, Lys599, Glu643, Leu644, Cys645, Ala646, Glu651, and Asp711^47^. Compound **11** showed highest binding affinity with the enzyme’s active site through Asp711, Lys599, and Ser710 amino acids (Fig. 9F). The carbonyl oxygen of the co-crystallized ligand of BDR4 interacts with Asn140 and the aminopyrimidine moiety interacts with Pro82^48^. Similarly, compound 12 showed the same interactions with Pro82 (Fig. 9G).

## Conclusions

The anti-MRSA activity of three root extracts of three medicinal plants *J. officinale*, *R. damascene* and *P. officinalis* roots was evaluated and revealed the strongest potency of *P. officinalis* roots. This is the first study of the antimicrobial evaluation of *P. officinalis* roots, therefore, untargeted metabolomics profiling of the root derived extract of *P. officinalis* was performed by using LC-HR-ESI-MS, in which twenty compounds of multivariate groups of secondary metabolites like terpenoids, terpenidal glycosides and phenolic compounds were dereplicated. The analysis of the molecular docking data provides valuable insights into the binding affinities and potential specificities of the 20 compounds against the 7 biological targets. Future experiments will include isolation of the bioactive compounds and test them against MSRA as well as study the detailed mechanism of action in vitro as well as in vivo. These findings can guide further investigations and contribute to the rational design and development of novel therapeutics or lead compounds in drug discovery research.

### Supplementary Information

Supplementary Tables.

## Data Availability

All data generated or analyzed during this study are included in this published article and its supplementary information files.
