# Supplementary material for: Capturing the antimicrobial profile of Paeonia officinalis, Jasminum officinale and Rosa damascene against methicillin resistant Staphylococcus aureus with metabolomics analysis and network pharmacology
Source: Sci Rep. 2024 Jun 13;14:13621. doi: 10.1038/s41598-024-62369-5 (PMC11176355; doi:10.1038/s41598-024-62369-5)
Supplement: Supplementary file 1 — Supplementary Tables. [file 41598_2024_62369_MOESM1_ESM.docx]

Capturing the antimicrobial profile of *Paeonia officinalis*, *J. officinale* and *R. damascene* against methicillin-resistant *Staphylococcus aureus* with metabolomics analysis and network pharmacology

Table S1: Metabolite-target network analysis of *Paeonia officinalis*

| Node name | Number Of Undirected Edges | Average Shortest PathLength | Betweenness Centrality | Closeness Centrality | Degree | Radiality |
| --- | --- | --- | --- | --- | --- | --- |
| cpd 1 | 27 | 2.900355872 | 0.099476199 | 0.344785276 | 27 | 0.977643 |
| ERN1 | 3 | 3.669039146 | 0.00212967 | 0.272550921 | 3 | 0.9686 |
| HMGCR | 4 | 3.412811388 | 0.020205275 | 0.293013556 | 4 | 0.971614 |
| CA9 | 6 | 2.978647687 | 0.020112597 | 0.33572282 | 6 | 0.976722 |
| EP300 | 1 | 3.896797153 | 0 | 0.256621005 | 1 | 0.96592 |
| CA2 | 5 | 2.886120996 | 0.019456787 | 0.34648582 | 5 | 0.97781 |
| CA1 | 5 | 2.886120996 | 0.019456787 | 0.34648582 | 5 | 0.97781 |
| CA7 | 6 | 2.508896797 | 0.051015083 | 0.39858156 | 6 | 0.982248 |
| CA12 | 7 | 2.658362989 | 0.040468709 | 0.376171352 | 7 | 0.98049 |
| CA14 | 8 | 2.430604982 | 0.070654318 | 0.411420205 | 8 | 0.983169 |
| MAOB | 1 | 3.896797153 | 0 | 0.256621005 | 1 | 0.96592 |
| FASN | 2 | 3.818505338 | 2.33E-04 | 0.261882572 | 2 | 0.966841 |
| CISD1 | 2 | 3.818505338 | 2.33E-04 | 0.261882572 | 2 | 0.966841 |
| ALPG | 2 | 3.818505338 | 2.33E-04 | 0.261882572 | 2 | 0.966841 |
| PLAA | 2 | 3.818505338 | 2.33E-04 | 0.261882572 | 2 | 0.966841 |
| MAOA | 1 | 3.896797153 | 0 | 0.256621005 | 1 | 0.96592 |
| MB | 2 | 3.818505338 | 2.33E-04 | 0.261882572 | 2 | 0.966841 |
| KCNMA1 | 1 | 3.896797153 | 0 | 0.256621005 | 1 | 0.96592 |
| PIM3 | 1 | 3.896797153 | 0 | 0.256621005 | 1 | 0.96592 |
| ACHE | 3 | 3.483985765 | 0.010738016 | 0.287027579 | 3 | 0.970777 |
| CDC25B | 1 | 3.896797153 | 0 | 0.256621005 | 1 | 0.96592 |
| NAT1 | 2 | 3.747330961 | 8.35E-04 | 0.2668566 | 2 | 0.967678 |
| GSK3B | 5 | 2.857651246 | 0.018530028 | 0.349937733 | 5 | 0.978145 |
| PLEC | 2 | 3.818505338 | 2.33E-04 | 0.261882572 | 2 | 0.966841 |
| CSNK1A1 | 2 | 3.818505338 | 2.33E-04 | 0.261882572 | 2 | 0.966841 |
| CSNK1D | 2 | 3.818505338 | 2.33E-04 | 0.261882572 | 2 | 0.966841 |
| PTPN22 | 1 | 3.896797153 | 0 | 0.256621005 | 1 | 0.96592 |
| EGLN1 | 1 | 3.896797153 | 0 | 0.256621005 | 1 | 0.96592 |
| cpd 2 | 53 | 2.565836299 | 0.267127531 | 0.389736477 | 53 | 0.981578 |
| AR | 1 | 3.56227758 | 0 | 0.280719281 | 1 | 0.969856 |
| NPC1L1 | 1 | 3.56227758 | 0 | 0.280719281 | 1 | 0.969856 |
| CYP19A1 | 1 | 3.56227758 | 0 | 0.280719281 | 1 | 0.969856 |
| G6PD | 1 | 3.56227758 | 0 | 0.280719281 | 1 | 0.969856 |
| GABBR1 | 1 | 3.56227758 | 0 | 0.280719281 | 1 | 0.969856 |
| NR1H4 | 1 | 3.56227758 | 0 | 0.280719281 | 1 | 0.969856 |
| GABRA2 | 1 | 3.56227758 | 0 | 0.280719281 | 1 | 0.969856 |
| GPBAR1 | 1 | 3.56227758 | 0 | 0.280719281 | 1 | 0.969856 |
| AKR1B10 | 1 | 3.56227758 | 0 | 0.280719281 | 1 | 0.969856 |
| FABP4 | 1 | 3.56227758 | 0 | 0.280719281 | 1 | 0.969856 |
| FABP3 | 1 | 3.56227758 | 0 | 0.280719281 | 1 | 0.969856 |
| FABP5 | 1 | 3.56227758 | 0 | 0.280719281 | 1 | 0.969856 |
| FABP2 | 1 | 3.56227758 | 0 | 0.280719281 | 1 | 0.969856 |
| KDM2A | 1 | 3.56227758 | 0 | 0.280719281 | 1 | 0.969856 |
| PHF8 | 1 | 3.56227758 | 0 | 0.280719281 | 1 | 0.969856 |
| PPARD | 1 | 3.56227758 | 0 | 0.280719281 | 1 | 0.969856 |
| SLC22A6 | 1 | 3.56227758 | 0 | 0.280719281 | 1 | 0.969856 |
| HSD11B1 | 3 | 3.320284698 | 0.005516993 | 0.301178992 | 3 | 0.972703 |
| MIF | 1 | 3.56227758 | 0 | 0.280719281 | 1 | 0.969856 |
| VDR | 4 | 2.807829181 | 0.020436399 | 0.356147022 | 4 | 0.978731 |
| UGT2B7 | 2 | 3.334519573 | 0.003615369 | 0.299893276 | 2 | 0.972535 |
| HAO1 | 1 | 3.56227758 | 0 | 0.280719281 | 1 | 0.969856 |
| CDC25A | 2 | 3.334519573 | 0.003615369 | 0.299893276 | 2 | 0.972535 |
| SERPINA6 | 1 | 3.56227758 | 0 | 0.280719281 | 1 | 0.969856 |
| SHBG | 3 | 3.320284698 | 0.010820947 | 0.301178992 | 3 | 0.972703 |
| HSD17B3 | 1 | 3.56227758 | 0 | 0.280719281 | 1 | 0.969856 |
| KDM5C | 1 | 3.56227758 | 0 | 0.280719281 | 1 | 0.969856 |
| GSR | 3 | 3.120996441 | 0.008161849 | 0.32041049 | 3 | 0.975047 |
| DRD2 | 6 | 2.715302491 | 0.032411118 | 0.368283093 | 6 | 0.97982 |
| LRRK2 | 2 | 3.334519573 | 0.003615369 | 0.299893276 | 2 | 0.972535 |
| PTGER2 | 1 | 3.56227758 | 0 | 0.280719281 | 1 | 0.969856 |
| PTGFR | 1 | 3.56227758 | 0 | 0.280719281 | 1 | 0.969856 |
| BRD4 | 1 | 3.56227758 | 0 | 0.280719281 | 1 | 0.969856 |
| BRD2 | 1 | 3.56227758 | 0 | 0.280719281 | 1 | 0.969856 |
| CYP11B1 | 1 | 3.56227758 | 0 | 0.280719281 | 1 | 0.969856 |
| CYP11B2 | 1 | 3.56227758 | 0 | 0.280719281 | 1 | 0.969856 |
| POLB | 2 | 3.419928826 | 0.00695471 | 0.292403746 | 2 | 0.97153 |
| PLG | 1 | 3.56227758 | 0 | 0.280719281 | 1 | 0.969856 |
| PTPN1 | 6 | 2.786476868 | 0.038787341 | 0.358876117 | 6 | 0.978983 |
| FFAR1 | 1 | 3.56227758 | 0 | 0.280719281 | 1 | 0.969856 |
| MPO | 2 | 3.334519573 | 0.003615369 | 0.299893276 | 2 | 0.972535 |
| PPARA | 1 | 3.56227758 | 0 | 0.280719281 | 1 | 0.969856 |
| CDC45 | 1 | 3.56227758 | 0 | 0.280719281 | 1 | 0.969856 |
| PTPRC | 1 | 3.56227758 | 0 | 0.280719281 | 1 | 0.969856 |
| CA3 | 1 | 3.56227758 | 0 | 0.280719281 | 1 | 0.969856 |
| CA6 | 3 | 2.814946619 | 0.010982704 | 0.355246523 | 3 | 0.978648 |
| CA13 | 4 | 2.743772242 | 0.015951112 | 0.364461738 | 4 | 0.979485 |
| CA5B | 1 | 3.56227758 | 0 | 0.280719281 | 1 | 0.969856 |
| cpd 3 | 18 | 3.106761566 | 0.070664551 | 0.32187858 | 18 | 0.975215 |
| SRD5A1 | 2 | 3.434163701 | 0.002265527 | 0.29119171 | 2 | 0.971363 |
| HDAC3 | 2 | 3.932384342 | 0.00155683 | 0.254298643 | 2 | 0.965501 |
| HDAC2 | 2 | 3.932384342 | 0.00155683 | 0.254298643 | 2 | 0.965501 |
| HDAC1 | 6 | 2.772241993 | 0.028713528 | 0.36071887 | 6 | 0.97915 |
| NOS2 | 1 | 4.103202847 | 0 | 0.243712056 | 1 | 0.963492 |
| CYP2A6 | 1 | 4.103202847 | 0 | 0.243712056 | 1 | 0.963492 |
| HDAC5 | 1 | 4.103202847 | 0 | 0.243712056 | 1 | 0.963492 |
| HDAC7 | 1 | 4.103202847 | 0 | 0.243712056 | 1 | 0.963492 |
| HDAC4 | 1 | 4.103202847 | 0 | 0.243712056 | 1 | 0.963492 |
| HDAC9 | 1 | 4.103202847 | 0 | 0.243712056 | 1 | 0.963492 |
| NOX4 | 1 | 4.103202847 | 0 | 0.243712056 | 1 | 0.963492 |
| PNMT | 1 | 4.103202847 | 0 | 0.243712056 | 1 | 0.963492 |
| cpd 4 | 26 | 3.071174377 | 0.090125067 | 0.325608343 | 26 | 0.975633 |
| BAD | 1 | 4.067615658 | 0 | 0.245844269 | 1 | 0.96391 |
| TUBB1 | 1 | 4.067615658 | 0 | 0.245844269 | 1 | 0.96391 |
| MCL1 | 3 | 3.206405694 | 0.006862693 | 0.311875694 | 3 | 0.974042 |
| BCL2 | 1 | 4.067615658 | 0 | 0.245844269 | 1 | 0.96391 |
| ALB | 1 | 4.067615658 | 0 | 0.245844269 | 1 | 0.96391 |
| ADAMTS5 | 1 | 4.067615658 | 0 | 0.245844269 | 1 | 0.96391 |
| PIK3CD | 1 | 4.067615658 | 0 | 0.245844269 | 1 | 0.96391 |
| PRKDC | 1 | 4.067615658 | 0 | 0.245844269 | 1 | 0.96391 |
| PIK3CG | 1 | 4.067615658 | 0 | 0.245844269 | 1 | 0.96391 |
| PIK3CA | 1 | 4.067615658 | 0 | 0.245844269 | 1 | 0.96391 |
| cpd 5 | 8 | 3.24911032 | 0.011147344 | 0.307776561 | 8 | 0.97354 |
| PPM1B | 3 | 3.362989324 | 0.002949969 | 0.297354497 | 3 | 0.9722 |
| PRKCA | 3 | 3.362989324 | 0.002949969 | 0.297354497 | 3 | 0.9722 |
| PIK3CA | 2 | 3.839857651 | 2.29E-04 | 0.260426321 | 2 | 0.96659 |
| PRKCD | 3 | 3.113879004 | 0.006164367 | 0.321142857 | 3 | 0.975131 |
| ABCB1 | 1 | 4.245551601 | 0 | 0.235540654 | 1 | 0.961817 |
| cpd 6 | 38 | 2.857651246 | 0.130752133 | 0.349937733 | 38 | 0.978145 |
| OPRM1 | 2 | 3.128113879 | 0.002562402 | 0.319681456 | 2 | 0.974963 |
| OPRK1 | 4 | 2.950177936 | 0.010052494 | 0.338962606 | 4 | 0.977057 |
| AOC3 | 1 | 3.854092527 | 0 | 0.259464451 | 1 | 0.966422 |
| PPP1CA | 1 | 3.854092527 | 0 | 0.259464451 | 1 | 0.966422 |
| SIRT2 | 1 | 3.854092527 | 0 | 0.259464451 | 1 | 0.966422 |
| TRPV4 | 2 | 3.128113879 | 0.002562402 | 0.319681456 | 2 | 0.974963 |
| PPP5C | 1 | 3.854092527 | 0 | 0.259464451 | 1 | 0.966422 |
| CES1 | 1 | 3.854092527 | 0 | 0.259464451 | 1 | 0.966422 |
| MMP2 | 1 | 3.854092527 | 0 | 0.259464451 | 1 | 0.966422 |
| PPP1CC | 2 | 3.377224199 | 0.001737775 | 0.296101159 | 2 | 0.972033 |
| DAO | 5 | 2.807829181 | 0.011139365 | 0.356147022 | 5 | 0.978731 |
| PRKCG | 2 | 3.128113879 | 0.002562402 | 0.319681456 | 2 | 0.974963 |
| PRKCE | 2 | 3.128113879 | 0.002562402 | 0.319681456 | 2 | 0.974963 |
| PRKCH | 2 | 3.128113879 | 0.002562402 | 0.319681456 | 2 | 0.974963 |
| PRKCQ | 2 | 3.128113879 | 0.002562402 | 0.319681456 | 2 | 0.974963 |
| AHCY | 5 | 2.786476868 | 0.013335612 | 0.358876117 | 5 | 0.978983 |
| CYP3A4 | 1 | 3.854092527 | 0 | 0.259464451 | 1 | 0.966422 |
| ERBB2 | 1 | 3.854092527 | 0 | 0.259464451 | 1 | 0.966422 |
| EGFR | 4 | 2.871886121 | 0.008488155 | 0.348203222 | 4 | 0.977978 |
| PTGS2 | 3 | 3.071174377 | 0.004593201 | 0.325608343 | 3 | 0.975633 |
| NFKBIA | 1 | 3.854092527 | 0 | 0.259464451 | 1 | 0.966422 |
| RELA | 1 | 3.854092527 | 0 | 0.259464451 | 1 | 0.966422 |
| PTGS1 | 2 | 3.505338078 | 0.001488496 | 0.285279188 | 2 | 0.970525 |
| EHMT2 | 1 | 3.854092527 | 0 | 0.259464451 | 1 | 0.966422 |
| PREP | 1 | 3.854092527 | 0 | 0.259464451 | 1 | 0.966422 |
| PSMB5 | 1 | 3.854092527 | 0 | 0.259464451 | 1 | 0.966422 |
| cpd 8 | 34 | 2.779359431 | 0.05212622 | 0.359795134 | 34 | 0.979066 |
| CDA | 9 | 2.786476868 | 0.008646929 | 0.358876117 | 9 | 0.978983 |
| HTR2B | 5 | 3.042704626 | 0.001545987 | 0.328654971 | 5 | 0.975968 |
| ADRA2A | 5 | 3.042704626 | 0.001545987 | 0.328654971 | 5 | 0.975968 |
| ADRA2C | 5 | 3.042704626 | 0.001545987 | 0.328654971 | 5 | 0.975968 |
| ADRA2B | 5 | 3.042704626 | 0.001545987 | 0.328654971 | 5 | 0.975968 |
| DRD1 | 5 | 3.042704626 | 0.001545987 | 0.328654971 | 5 | 0.975968 |
| ADRA1D | 5 | 3.042704626 | 0.001545987 | 0.328654971 | 5 | 0.975968 |
| HTR2A | 5 | 3.042704626 | 0.001545987 | 0.328654971 | 5 | 0.975968 |
| HTR2C | 5 | 3.042704626 | 0.001545987 | 0.328654971 | 5 | 0.975968 |
| DRD3 | 5 | 3.042704626 | 0.001545987 | 0.328654971 | 5 | 0.975968 |
| CYP2D6 | 5 | 3.042704626 | 0.001545987 | 0.328654971 | 5 | 0.975968 |
| HTR6 | 5 | 3.042704626 | 0.001545987 | 0.328654971 | 5 | 0.975968 |
| ADRA1A | 5 | 3.042704626 | 0.001545987 | 0.328654971 | 5 | 0.975968 |
| HTR1B | 5 | 3.042704626 | 0.001545987 | 0.328654971 | 5 | 0.975968 |
| RORC | 6 | 2.93594306 | 0.003150376 | 0.340606061 | 6 | 0.977224 |
| TYMS | 5 | 2.964412811 | 0.002818858 | 0.337334934 | 5 | 0.976889 |
| TK1 | 6 | 2.836298932 | 0.004333456 | 0.352572146 | 6 | 0.978396 |
| OGA | 8 | 2.800711744 | 0.007111694 | 0.357052097 | 8 | 0.978815 |
| FUCA1 | 7 | 2.822064057 | 0.005514572 | 0.354350567 | 7 | 0.978564 |
| ADA | 9 | 2.786476868 | 0.008646929 | 0.358876117 | 9 | 0.978983 |
| ADK | 9 | 2.743772242 | 0.012515837 | 0.364461738 | 9 | 0.979485 |
| CDK9 | 2 | 3.320284698 | 2.37E-04 | 0.301178992 | 2 | 0.972703 |
| CSNK2A1 | 5 | 2.93594306 | 0.002513764 | 0.340606061 | 5 | 0.977224 |
| STAT3 | 5 | 3.15658363 | 0.001751394 | 0.316798196 | 5 | 0.974628 |
| HEXA | 1 | 3.775800712 | 0 | 0.264844486 | 1 | 0.967344 |
| HEXB | 1 | 3.775800712 | 0 | 0.264844486 | 1 | 0.967344 |
| KDM4C | 3 | 3.270462633 | 6.66E-04 | 0.305767138 | 3 | 0.973289 |
| PTGES | 3 | 3.128113879 | 9.85E-04 | 0.319681456 | 3 | 0.974963 |
| cpd 9 | 67 | 2.480427046 | 0.171061874 | 0.403156385 | 67 | 0.982583 |
| GBA | 3 | 3.24911032 | 5.90E-04 | 0.307776561 | 3 | 0.97354 |
| GAA | 3 | 3.128113879 | 5.07E-04 | 0.319681456 | 3 | 0.974963 |
| CDK2 | 1 | 3.476868327 | 0 | 0.287615148 | 1 | 0.97086 |
| ADORA1 | 6 | 2.950177936 | 0.003536096 | 0.338962606 | 6 | 0.977057 |
| GAPDH | 4 | 2.971530249 | 0.001535227 | 0.336526946 | 4 | 0.976806 |
| ADORA2A | 9 | 2.75088968 | 0.01258317 | 0.363518758 | 9 | 0.979401 |
| ADORA3 | 8 | 2.786476868 | 0.01028849 | 0.358876117 | 8 | 0.978983 |
| HK2 | 3 | 3.078291815 | 5.62E-04 | 0.324855491 | 3 | 0.97555 |
| HK1 | 3 | 3.078291815 | 5.62E-04 | 0.324855491 | 3 | 0.97555 |
| TYR | 4 | 3.234875445 | 0.001092825 | 0.309130913 | 4 | 0.973707 |
| MAPK1 | 4 | 3 | 0.001885935 | 0.333333333 | 4 | 0.976471 |
| MANBA | 3 | 3.078291815 | 5.62E-04 | 0.324855491 | 3 | 0.97555 |
| HSPA8 | 5 | 2.871886121 | 0.003178051 | 0.348203222 | 5 | 0.977978 |
| CDK1 | 4 | 2.985765125 | 0.00190607 | 0.334922527 | 4 | 0.976638 |
| PNP | 7 | 2.793594306 | 0.00850468 | 0.357961783 | 7 | 0.978899 |
| SLC5A2 | 4 | 3.049822064 | 0.001217478 | 0.327887981 | 4 | 0.975884 |
| HSPA5 | 5 | 2.871886121 | 0.003178051 | 0.348203222 | 5 | 0.977978 |
| F2 | 3 | 3.24911032 | 5.90E-04 | 0.307776561 | 3 | 0.97354 |
| GRK1 | 3 | 3.056939502 | 6.22E-04 | 0.327124563 | 3 | 0.975801 |
| SLC5A1 | 3 | 3.056939502 | 6.22E-04 | 0.327124563 | 3 | 0.975801 |
| DPP4 | 3 | 3.071174377 | 0.001069556 | 0.325608343 | 3 | 0.975633 |
| LGALS3 | 7 | 2.871886121 | 0.00768009 | 0.348203222 | 7 | 0.977978 |
| LGALS9 | 3 | 3.348754448 | 0.001177973 | 0.298618491 | 3 | 0.972368 |
| MMP3 | 5 | 2.850533808 | 0.012565726 | 0.350811486 | 5 | 0.978229 |
| MMP9 | 3 | 3.227758007 | 0.004262126 | 0.309812569 | 3 | 0.973791 |
| ADAM17 | 5 | 2.850533808 | 0.012565726 | 0.350811486 | 5 | 0.978229 |
| MMP1 | 5 | 2.850533808 | 0.012565726 | 0.350811486 | 5 | 0.978229 |
| SLC5A4 | 2 | 3.348754448 | 9.45E-05 | 0.298618491 | 2 | 0.972368 |
| CDK4 | 2 | 3.348754448 | 9.45E-05 | 0.298618491 | 2 | 0.972368 |
| SLC29A1 | 3 | 3.056939502 | 6.22E-04 | 0.327124563 | 3 | 0.975801 |
| PYGL | 1 | 3.476868327 | 0 | 0.287615148 | 1 | 0.97086 |
| PIM1 | 1 | 3.476868327 | 0 | 0.287615148 | 1 | 0.97086 |
| ADORA2B | 2 | 3.348754448 | 9.45E-05 | 0.298618491 | 2 | 0.972368 |
| AKR1C1 | 1 | 3.476868327 | 0 | 0.287615148 | 1 | 0.97086 |
| GLRA1 | 3 | 3.078291815 | 5.62E-04 | 0.324855491 | 3 | 0.97555 |
| GLRA2 | 3 | 3.078291815 | 5.62E-04 | 0.324855491 | 3 | 0.97555 |
| EDNRA | 2 | 3.135231317 | 2.55E-04 | 0.318955732 | 2 | 0.97488 |
| CA4 | 2 | 3.135231317 | 2.55E-04 | 0.318955732 | 2 | 0.97488 |
| CA5A | 2 | 3.135231317 | 2.55E-04 | 0.318955732 | 2 | 0.97488 |
| IDO1 | 2 | 3.348754448 | 9.45E-05 | 0.298618491 | 2 | 0.972368 |
| MME | 2 | 3.135231317 | 2.55E-04 | 0.318955732 | 2 | 0.97488 |
| TYMP | 4 | 2.985765125 | 0.00301741 | 0.334922527 | 4 | 0.976638 |
| cpd 11 | 55 | 2.551601423 | 0.099498477 | 0.391910739 | 55 | 0.981746 |
| EPHX2 | 2 | 3.441281139 | 1.47E-04 | 0.290589452 | 2 | 0.971279 |
| CDK2 | 5 | 2.950177936 | 0.002455489 | 0.338962606 | 5 | 0.977057 |
| SLC28A3 | 1 | 3.548042705 | 0 | 0.281845537 | 1 | 0.970023 |
| ESR1 | 1 | 3.548042705 | 0 | 0.281845537 | 1 | 0.970023 |
| CDK9 | 3 | 3.06405694 | 8.37E-04 | 0.326364692 | 3 | 0.975717 |
| AKR1C3 | 5 | 2.950177936 | 0.002455489 | 0.338962606 | 5 | 0.977057 |
| AKR1B1 | 3 | 3.234875445 | 0.001613785 | 0.309130913 | 3 | 0.973707 |
| PIN1 | 3 | 3.170818505 | 8.72E-04 | 0.315375982 | 3 | 0.974461 |
| DTYMK | 1 | 3.548042705 | 0 | 0.281845537 | 1 | 0.970023 |
| GSTP1 | 2 | 3.149466192 | 2.73E-04 | 0.317514124 | 2 | 0.974712 |
| GSTM2 | 2 | 3.149466192 | 2.73E-04 | 0.317514124 | 2 | 0.974712 |
| FOLH1 | 6 | 3.035587189 | 0.003281346 | 0.329425557 | 6 | 0.976052 |
| cpd 12 | 20 | 2.93594306 | 0.053316004 | 0.340606061 | 20 | 0.977224 |
| SQLE | 2 | 3.747330961 | 6.78E-04 | 0.2668566 | 2 | 0.967678 |
| F10 | 1 | 3.932384342 | 0 | 0.254298643 | 1 | 0.965501 |
| AMY1A | 1 | 3.932384342 | 0 | 0.254298643 | 1 | 0.965501 |
| SERPINE1 | 2 | 3.747330961 | 6.78E-04 | 0.2668566 | 2 | 0.967678 |
| BACE1 | 2 | 3.747330961 | 6.78E-04 | 0.2668566 | 2 | 0.967678 |
| HSP90AA1 | 3 | 3.163701068 | 0.001901151 | 0.316085489 | 3 | 0.974545 |
| PTPN2 | 1 | 3.932384342 | 0 | 0.254298643 | 1 | 0.965501 |
| TDP1 | 1 | 3.932384342 | 0 | 0.254298643 | 1 | 0.965501 |
| cpd 14 | 21 | 3.163701068 | 0.074457978 | 0.316085489 | 21 | 0.974545 |
| HDAC6 | 1 | 4.160142349 | 0 | 0.24037639 | 1 | 0.962822 |
| ADAMTS4 | 1 | 4.160142349 | 0 | 0.24037639 | 1 | 0.962822 |
| HDAC8 | 1 | 4.160142349 | 0 | 0.24037639 | 1 | 0.962822 |
| ALPL | 1 | 4.160142349 | 0 | 0.24037639 | 1 | 0.962822 |
| GRK6 | 1 | 4.160142349 | 0 | 0.24037639 | 1 | 0.962822 |
| JAK3 | 1 | 4.160142349 | 0 | 0.24037639 | 1 | 0.962822 |
| JAK1 | 1 | 4.160142349 | 0 | 0.24037639 | 1 | 0.962822 |
| MMP14 | 1 | 4.160142349 | 0 | 0.24037639 | 1 | 0.962822 |
| cpd 15 | 46 | 2.807829181 | 0.087551452 | 0.356147022 | 46 | 0.978731 |
| PPP2CA | 1 | 3.804270463 | 0 | 0.262862488 | 1 | 0.967009 |
| PPP2R5A | 1 | 3.804270463 | 0 | 0.262862488 | 1 | 0.967009 |
| HSD11B2 | 1 | 3.804270463 | 0 | 0.262862488 | 1 | 0.967009 |
| ATP1A1 | 1 | 3.804270463 | 0 | 0.262862488 | 1 | 0.967009 |
| BCL2L1 | 1 | 3.804270463 | 0 | 0.262862488 | 1 | 0.967009 |
| PTAFR | 4 | 3.035587189 | 0.001432551 | 0.329425557 | 4 | 0.976052 |
| FBP1 | 2 | 3.526690391 | 3.21E-04 | 0.283551968 | 2 | 0.970274 |
| IARS | 1 | 3.804270463 | 0 | 0.262862488 | 1 | 0.967009 |
| cpd 16 | 17 | 3.007117438 | 0.019820128 | 0.332544379 | 17 | 0.976387 |
| MGAM | 1 | 4.003558719 | 0 | 0.249777778 | 1 | 0.964664 |
| cpd 17 | 60 | 2.879003559 | 0.098732334 | 0.347342398 | 60 | 0.977894 |
| PPM1A | 3 | 3.149466192 | 4.79E-04 | 0.317514124 | 3 | 0.974712 |
| VEGFA | 4 | 3.113879004 | 9.15E-04 | 0.321142857 | 4 | 0.975131 |
| FGF1 | 4 | 3.113879004 | 9.15E-04 | 0.321142857 | 4 | 0.975131 |
| FGF2 | 4 | 3.113879004 | 9.15E-04 | 0.321142857 | 4 | 0.975131 |
| HPSE | 3 | 3.619217082 | 1.56E-04 | 0.276302852 | 3 | 0.969186 |
| PSEN2 | 3 | 3.619217082 | 1.56E-04 | 0.276302852 | 3 | 0.969186 |
| MLNR | 3 | 3.149466192 | 4.79E-04 | 0.317514124 | 3 | 0.974712 |
| LGALS4 | 2 | 3.825622776 | 6.92E-05 | 0.261395349 | 2 | 0.966757 |
| LGALS8 | 2 | 3.825622776 | 6.92E-05 | 0.261395349 | 2 | 0.966757 |
| PYGM | 2 | 3.669039146 | 3.83E-05 | 0.272550921 | 2 | 0.9686 |
| CASP3 | 1 | 3.87544484 | 0 | 0.258034894 | 1 | 0.966171 |
| CASP6 | 1 | 3.87544484 | 0 | 0.258034894 | 1 | 0.966171 |
| CASP7 | 1 | 3.87544484 | 0 | 0.258034894 | 1 | 0.966171 |
| CASP8 | 1 | 3.87544484 | 0 | 0.258034894 | 1 | 0.966171 |
| CASP2 | 1 | 3.87544484 | 0 | 0.258034894 | 1 | 0.966171 |
| AKR1C2 | 1 | 3.87544484 | 0 | 0.258034894 | 1 | 0.966171 |
| TREH | 3 | 3.619217082 | 1.56E-04 | 0.276302852 | 3 | 0.969186 |
| KCNH2 | 2 | 3.669039146 | 3.83E-05 | 0.272550921 | 2 | 0.9686 |
| cpd 18 | 85 | 2.338078292 | 0.247469495 | 0.427701674 | 85 | 0.984258 |
| PSEN2 | 1 | 3.334519573 | 0 | 0.299893276 | 1 | 0.972535 |
| MAPK10 | 1 | 3.334519573 | 0 | 0.299893276 | 1 | 0.972535 |
| FDFT1 | 2 | 3.263345196 | 9.41E-05 | 0.306434024 | 2 | 0.973372 |
| MMP13 | 1 | 3.334519573 | 0 | 0.299893276 | 1 | 0.972535 |
| MMP7 | 1 | 3.334519573 | 0 | 0.299893276 | 1 | 0.972535 |
| MMP8 | 1 | 3.334519573 | 0 | 0.299893276 | 1 | 0.972535 |
| PRKCB | 1 | 3.334519573 | 0 | 0.299893276 | 1 | 0.972535 |
| TLR9 | 1 | 3.334519573 | 0 | 0.299893276 | 1 | 0.972535 |
| PTPN11 | 1 | 3.334519573 | 0 | 0.299893276 | 1 | 0.972535 |
| cpd 19 | 38 | 3.149466192 | 0.039510369 | 0.317514124 | 38 | 0.974712 |
| TRPV1 | 1 | 4.145907473 | 0 | 0.241201717 | 1 | 0.962989 |
| AMY2A | 1 | 4.145907473 | 0 | 0.241201717 | 1 | 0.962989 |
| P2RX3 | 1 | 4.145907473 | 0 | 0.241201717 | 1 | 0.962989 |
| cpd 20 | 50 | 2.814946619 | 0.0452208 | 0.355246523 | 50 | 0.978648 |
| cpd 7 | 0 | 0 | 0 | 0 | 0 | Infinity |
| cpd 10 | 0 | 0 | 0 | 0 | 0 | Infinity |
| cpd 13 | 0 | 0 | 0 | 0 | 0 | Infinity |

Table S2: the top 20 gene enrichment analysis in the terms of biological process, cellular component and molecular functions, each term was arranged in descending order according to no. of percentage of genes

| **No.** |  | **Percentage of genes** | **Fold enrichment** | **genes mapped (from input data set)** |
| --- | --- | --- | --- | --- |
| **Biological process** | | | | |
| 1 | Signal transduction | 31.66023166 | 1.458710515 | ERN1; PLAA; PIM3; CDC25B; CSNK1D; PTPN22; AR; GABBR1; NR1H4; GPBAR1; FABP4; MIF; CDC25A; DRD2; PTGER2; PTGFR; BRD4; PTPN1; FFAR1; PTPRC; MCL1; PIK3CD; PRKDC; PIK3CG; PIK3CA; PPM1B; PRKCA; PRKCD; OPRM1; OPRK1; SIRT2; TRPV4; PPP5C; PPP1CC; PRKCG; PRKCE; PRKCH; PRKCQ; ERBB2; EGFR; HTR2B; ADRA2A; ADRA2C; ADRA2B; DRD1; ADRA1D; HTR2A; HTR2C; DRD3; HTR6; ADRA1A; HTR1B; CSNK2A1; ADORA1; ADORA2A; ADORA3; MAPK1; CDK1; CDK4; PIM1; ADORA2B; EDNRA; TYMP; PTPN2; GRK6; JAK3; JAK1; PPP2CA; PPP2R5A; PTAFR; PPM1A; VEGFA; FGF1; FGF2; MLNR; LGALS4; LGALS8; MAPK10; PRKCB; TLR9; PTPN11; TRPV1; |
| 2 | Metabolism | 29.72972973 | 3.201829401 | HMGCR; CA9; CA2; CA1; CA7; CA12; CA14; MAOB; FASN; MAOA; ACHE; NAT1; GSK3B; G6PD; NR1H4; AKR1B10; HSD11B1; UGT2B7; HAO1; HSD17B3; GSR; CYP11B1; CYP11B2; MPO; CA3; CA6; CA13; CA5B; SRD5A1; NOS2; CYP2A6; NOX4; PNMT; CES1; DAO; AHCY; CYP3A4; PTGS2; CDA; TYMS; TK1; FUCA1; ADA; HEXA; HEXB; GAA; GAPDH; HK2; HK1; TYR; MANBA; PNP; GRK1; PYGL; AKR1C1; CA4; CA5A; IDO1; EPHX2; AKR1C3; AKR1B1; PIN1; DTYMK; GSTP1; GSTM2; SQLE; AMY1A; ALPL; HSD11B2; FBP1; MGAM; HPSE; PYGM; AKR1C2; TREH; FDFT1; AMY2A; |
| 3 | Energy pathways | 27.7992278 | 3.085614935 | HMGCR; CA9; CA2; CA1; CA12; CA14; MAOB; FASN; MAOA; ACHE; NAT1; GSK3B; G6PD; AKR1B10; UGT2B7; HAO1; HSD17B3; GSR; CYP11B1; CYP11B2; MPO; CA3; CA6; CA13; CA5B; SRD5A1; NOS2; CYP2A6; NOX4; PNMT; DAO; AHCY; CYP3A4; PTGS2; CDA; TYMS; TK1; FUCA1; ADA; HEXA; HEXB; GAA; GAPDH; HK2; HK1; TYR; MANBA; PNP; GRK1; PYGL; AKR1C1; CA4; CA5A; IDO1; EPHX2; AKR1C3; AKR1B1; PIN1; DTYMK; GSTP1; GSTM2; SQLE; AMY1A; ALPL; HSD11B2; FBP1; MGAM; HPSE; PYGM; TREH; FDFT1; AMY2A; |
| 4 | Cell communication | 27.02702703 | 1.31938554 | ERN1; PLAA; PIM3; CDC25B; CSNK1D; AR; GABBR1; NR1H4; GPBAR1; FABP4; MIF; CDC25A; DRD2; PTGER2; PTGFR; BRD4; PTPN1; FFAR1; PTPRC; PRKDC; PIK3CG; PIK3CA; PRKCA; PRKCD; OPRK1; SIRT2; PPP5C; PPP1CC; PRKCG; PRKCE; PRKCH; PRKCQ; ERBB2; EGFR; HTR2B; ADRA2A; ADRA2C; ADRA2B; DRD1; ADRA1D; HTR2A; HTR2C; DRD3; HTR6; ADRA1A; HTR1B; ADORA1; ADORA2A; ADORA3; MAPK1; CDK1; CDK4; PIM1; ADORA2B; EDNRA; TYMP; PTPN2; GRK6; JAK3; JAK1; PPP2CA; PPP2R5A; PTAFR; VEGFA; FGF1; FGF2; MLNR; LGALS4; PTPN11; TRPV1; |
| 5 | Protein metabolism | 10.81081081 | 1.481452541 | EGLN1; KDM2A; PLG; ADAMTS5; MMP2; PREP; PSMB5; HSPA8; HSPA5; F2; DPP4; MMP3; MMP9; ADAM17; MMP1; MME; FOLH1; F10; SERPINE1; BACE1; HSP90AA1; ADAMTS4; MMP14; IARS; PSEN2; MMP13; MMP7; MMP8; |
| 6 | Regulation of nucleobase, nucleoside, nucleotide and nucleic acid metabolism | 10.03861004 | 0.643572047 | EP300; PHF8; KDM5C; BRD2; POLB; PPARA; CDC45; HDAC3; HDAC2; HDAC1; HDAC5; HDAC7; HDAC4; NFKBIA; RELA; EHMT2; RORC; ADK; CDK9; STAT3; KDM4C; CDK2; LGALS3; ESR1; HDAC6; HDAC8; |
| 7 | Transport | 8.88030888 | 1.325178947 | MB; KCNMA1; NPC1L1; GABRA2; FABP3; FABP5; FABP2; SLC22A6; SERPINA6; SHBG; ALB; ABCB1; SLC5A2; SLC5A1; SLC5A4; SLC29A1; GLRA1; GLRA2; SLC28A3; ATP1A1; AKR1C2; KCNH2; P2RX3; |
| 8 | Apoptosis | 4.247104247 | 3.501730019 | BAD; MCL1; BCL2; LGALS9; BCL2L1; PSEN2; CASP3; CASP6; CASP7; CASP8; CASP2; |
| 9 | Regulation of gene expression, epigenetic | 1.158301158 | 3.190762829 | VDR; HDAC7; HDAC9; |
| 10 | Lipid metabolism | 1.158301158 | 6.380559053 | PTGS1; PTGES; GBA; |
| 11 | Biological_process unknown | 0.772200772 | 0.029728976 | CISD1; LRRK2; |
| 12 | Immune response | 0.772200772 | 0.244176461 | AOC3; LGALS9; |
| 13 | Anti-apoptosis | 0.772200772 | 4.393879521 | MCL1; RORC; |
| 14 | Cell cycle | 0.772200772 | 6.390190071 | CSNK1A1; BAD; |
| 15 | Protein modification | 0.772200772 | 7.028889729 | EHMT2; CSNK2A1; |
| 16 | Inflammatory response | 0.772200772 | 10.03912088 | LGALS9; TRPV1; |
| 17 | Cell growth and/or maintenance | 0.386100386 | 0.062820697 | TUBB1; |
| 18 | Neurotransmitter metabolism | 0.386100386 | 35.16115054 | ACHE; |
| 19 | Regulation of cell cycle | 0.386100386 | 1.239675716 | ADRA1A; |
| 20 | Ion transport | 0.386100386 | 1.472066498 | KCNH2; |
| cellular component | | | | |
| 1 | Cytoplasm | 58.13953488 | 1.488612518 | CA9; EP300; CA2; CA1; CA7; FASN; PLAA; PIM3; CDC25B; NAT1; GSK3B; PLEC; CSNK1A1; CSNK1D; PTPN22; EGLN1; AR; CYP19A1; G6PD; GPBAR1; FABP4; FABP3; FABP5; FABP2; MIF; VDR; CDC25A; KDM5C; GSR; LRRK2; BRD4; BRD2; POLB; PTPN1; MPO; PPARA; CDC45; CA3; CA6; CA13; HDAC3; HDAC2; HDAC1; NOS2; HDAC5; HDAC7; HDAC4; HDAC9; BAD; TUBB1; MCL1; BCL2; ALB; PIK3CD; PRKDC; PIK3CG; PPM1B; PRKCA; PRKCD; PPP1CA; SIRT2; PPP5C; PPP1CC; PRKCG; PRKCE; PRKCH; PRKCQ; AHCY; CYP3A4; ERBB2; EGFR; PTGS2; NFKBIA; RELA; PTGS1; EHMT2; PREP; PSMB5; CDA; HTR2B; ADRA2A; ADRA2C; TYMS; TK1; ADA; ADK; CDK9; CSNK2A1; STAT3; CDK2; ADORA1; GAPDH; ADORA2A; ADORA3; HK1; TYR; MAPK1; HSPA8; CDK1; PNP; HSPA5; GRK1; LGALS3; LGALS9; CDK4; PYGL; PIM1; AKR1C1; IDO1; MME; TYMP; EPHX2; ESR1; AKR1C3; AKR1B1; PIN1; DTYMK; GSTP1; GSTM2; FOLH1; SERPINE1; HSP90AA1; PTPN2; TDP1; HDAC6; HDAC8; ALPL; GRK6; JAK3; JAK1; PPP2CA; PPP2R5A; BCL2L1; FBP1; IARS; PPM1A; VEGFA; FGF1; HPSE; LGALS4; LGALS8; CASP3; CASP6; CASP7; CASP8; CASP2; MAPK10; PRKCB; TLR9; PTPN11; |
| 2 | Nucleus | 39.53488372 | 0.984068205 | ERN1; EP300; CA2; FASN; CDC25B; GSK3B; PLEC; CSNK1A1; CSNK1D; PTPN22; AR; GABBR1; NR1H4; KDM2A; PHF8; PPARD; VDR; UGT2B7; CDC25A; KDM5C; BRD4; BRD2; POLB; PTPN1; MPO; PPARA; CDC45; SRD5A1; HDAC3; HDAC2; HDAC1; HDAC5; HDAC7; HDAC4; HDAC9; NOX4; TUBB1; MCL1; BCL2; ALB; PRKDC; PIK3CG; PRKCA; PRKCD; PPP1CA; SIRT2; PPP5C; PPP1CC; PRKCG; PRKCQ; ERBB2; EGFR; PTGS2; NFKBIA; RELA; EHMT2; PREP; PSMB5; CDA; ADRA2A; RORC; TYMS; CDK9; CSNK2A1; STAT3; KDM4C; CDK2; GAPDH; MAPK1; HSPA8; CDK1; PNP; HSPA5; LGALS3; ADAM17; CDK4; PIM1; TYMP; ESR1; AKR1C3; PIN1; GSTP1; HSP90AA1; PTPN2; TDP1; HDAC6; HDAC8; JAK1; PPP2CA; BCL2L1; PPM1A; FGF1; FGF2; HPSE; LGALS8; CASP3; CASP6; CASP7; CASP8; CASP2; MAPK10; PRKCB; |
| 3 | Plasma membrane | 37.59689922 | 1.572813096 | CA9; CA2; CA12; CA14; ACHE; PLEC; CSNK1D; PTPN22; AR; NPC1L1; GABBR1; GPBAR1; SLC22A6; SERPINA6; DRD2; PTGER2; PTGFR; FFAR1; PTPRC; NOS2; NOX4; PIK3CG; PIK3CA; PRKCA; PRKCD; ABCB1; OPRM1; AOC3; TRPV4; PPP5C; MMP2; PRKCG; PRKCE; PRKCH; PRKCQ; ERBB2; EGFR; PTGS2; PTGS1; HTR2B; ADRA2B; DRD1; ADRA1D; HTR2A; HTR2C; DRD3; HTR6; HTR1B; FUCA1; ADA; CSNK2A1; ADORA1; ADORA2A; ADORA3; HK1; SLC5A2; HSPA5; F2; SLC5A1; DPP4; LGALS3; LGALS9; MMP9; ADAM17; SLC5A4; SLC29A1; PIM1; ADORA2B; GLRA1; GLRA2; EDNRA; CA4; MME; SLC28A3; ESR1; FOLH1; F10; SERPINE1; BACE1; ALPL; GRK6; MMP14; ATP1A1; PTAFR; MGAM; FGF2; PSEN2; MLNR; LGALS4; CASP3; TREH; KCNH2; PRKCB; TLR9; PTPN11; TRPV1; P2RX3; |
| 4 | Exosomes | 23.25581395 | 1.656797473 | CA2; FASN; PLEC; G6PD; AKR1B10; FABP3; SLC22A6; MIF; GSR; LRRK2; PLG; MPO; PTPRC; CA6; ALB; PRKDC; PRKCA; PRKCD; ABCB1; PPP1CA; PRKCH; AHCY; ERBB2; EGFR; PTGS1; PSMB5; FUCA1; ADK; GAA; GAPDH; HSPA8; CDK1; PNP; SLC5A2; HSPA5; SLC5A1; DPP4; LGALS3; SLC29A1; PYGL; CA4; MME; EPHX2; AKR1B1; GSTP1; GSTM2; FOLH1; AMY1A; HSP90AA1; ALPL; PPP2CA; ATP1A1; FBP1; IARS; MGAM; LGALS4; TREH; MMP7; PRKCB; AMY2A; |
| 5 | Extracellular | 21.70542636 | 1.731077905 | HMGCR; CA2; ACHE; AR; G6PD; GABBR1; FABP3; MIF; SERPINA6; SHBG; PLG; MPO; CA3; CA6; ALB; ADAMTS5; PIK3CA; CES1; MMP2; ERBB2; EGFR; CDA; ADRA1D; ADA; STAT3; HEXB; GAPDH; MANBA; F2; GRK1; SLC5A1; DPP4; LGALS3; LGALS9; MMP3; MMP9; MMP1; TYMP; AKR1B1; SQLE; F10; AMY1A; SERPINE1; ADAMTS4; ALPL; MMP14; VEGFA; FGF1; FGF2; LGALS8; PYGM; FDFT1; MMP13; MMP7; MMP8; AMY2A; |
| 6 | Cytosol | 20.93023256 | 2.586077223 | FASN; CDC25B; NAT1; GSK3B; PLEC; CSNK1A1; CSNK1D; EGLN1; G6PD; GSR; PTPN1; HDAC1; NOS2; PNMT; BAD; BCL2; PRKDC; PIK3CG; PIK3CA; PRKCA; PRKCD; PPP1CA; PRKCG; NFKBIA; RELA; PSMB5; CDA; ADA; CDK2; MAPK1; HSPA8; CDK1; CDK4; AKR1C1; IDO1; EPHX2; AKR1B1; DTYMK; HSP90AA1; JAK1; PPP2CA; BCL2L1; IARS; FGF1; LGALS4; PYGM; CASP3; CASP6; CASP7; CASP8; CASP2; AKR1C2; PRKCB; PTPN11; |
| 7 | Lysosome | 16.27906977 | 1.462686575 | CA2; CA1; MAOA; PLEC; CYP19A1; GSR; MPO; ALB; ABCB1; AOC3; AHCY; ERBB2; EGFR; FUCA1; ADA; ADK; CSNK2A1; HEXA; HEXB; GBA; GAA; GAPDH; HK1; TYR; MANBA; HSPA8; PNP; HSPA5; DPP4; LGALS3; MME; TYMP; AKR1B1; GSTP1; BACE1; HSP90AA1; PPP2CA; HSD11B2; ATP1A1; MGAM; HPSE; FDFT1; |
| 8 | Mitochondrion | 14.72868217 | 1.702883663 | MAOB; FASN; MAOA; PLEC; GSR; CYP11B1; CYP11B2; PTPRC; CA5B; HDAC1; BAD; MCL1; BCL2; PRKDC; PRKCD; PPP1CA; NFKBIA; PREP; CYP2D6; TYMS; STAT3; GAPDH; HK2; HK1; HSPA8; CDK1; HSPA5; CA5A; GSTP1; HSP90AA1; PPP2CA; ATP1A1; BCL2L1; IARS; MGAM; CASP3; CASP8; CASP2; |
| 9 | Endoplasmic reticulum | 14.72868217 | 1.941963896 | ERN1; HMGCR; CYP19A1; G6PD; FABP5; HSD11B1; UGT2B7; HSD17B3; PTPN1; MPO; SRD5A1; CYP2A6; NOX4; BCL2; PRKCA; PRKCD; OPRM1; TRPV4; PRKCE; CYP3A4; PTGS2; PTGS1; CYP2D6; HEXB; TYR; HSPA5; MME; SQLE; BACE1; PTPN2; HPSE; PSEN2; PYGM; CASP7; KCNH2; FDFT1; TLR9; TRPV1; |
| 10 | Integral to plasma membrane | 13.95348837 | 2.174642565 | GABBR1; SLC22A6; DRD2; PTGER2; PTGFR; FFAR1; OPRM1; OPRK1; ADRA2A; ADRA2C; ADRA2B; ADRA1D; HTR2A; DRD3; HTR6; ADRA1A; HTR1B; ADORA1; ADORA2A; ADORA3; SLC5A1; ADAM17; SLC29A1; ADORA2B; GLRA1; GLRA2; EDNRA; MME; FOLH1; BACE1; MMP14; PTAFR; PSEN2; MLNR; TRPV1; P2RX3; |
| 11 | Nucleolus | 9.689922481 | 1.122254222 | EP300; PLEC; CSNK1A1; CSNK1D; KDM2A; PHF8; BRD4; HDAC2; HDAC1; TUBB1; PRKDC; PPP1CA; PPP1CC; TYMS; CDK9; CSNK2A1; STAT3; CDK2; GAPDH; MAPK1; HSPA8; CDK1; HSPA5; CASP3; CASP8; |
| 12 | Integral to membrane | 8.914728682 | 0.762554345 | CA9; CA12; CA14; KCNMA1; ACHE; NPC1L1; NOX4; ABCB1; OPRK1; AOC3; TRPV4; ERBB2; ADRA2A; ADRA2C; ADRA1A; PTGES; SLC5A2; GLRA1; SLC28A3; BACE1; PTAFR; FDFT1; TRPV1; |
| 13 | Extracellular space | 7.364341085 | 2.654032832 | PLG; MPO; ALB; MMP2; EGFR; F2; MMP3; MMP9; AKR1B1; SERPINE1; HSP90AA1; ADAMTS4; VEGFA; FGF2; LGALS8; MMP13; MMP7; MMP8; AMY2A; |
| 14 | Membrane | 6.976744186 | 2.902348433 | ACHE; HSD11B1; MCL1; BCL2; AOC3; EGFR; ADA; PTGES; DPP4; EDNRA; MME; ESR1; FOLH1; ALPL; MMP14; PPP2CA; ATP1A1; PTAFR; |
| 15 | Membrane fraction | 6.589147287 | 2.772885598 | CYP19A1; SLC22A6; UGT2B7; LRRK2; PRKCA; ABCB1; PRKCG; CYP3A4; DRD1; PTGES; ADORA2A; SLC29A1; CA4; FOLH1; PPP2R5A; ATP1A1; CASP8; |
| 16 | Golgi aparatus | 6.201550388 | 1.006723801 | CA2; FASN; ACHE; CSNK1D; OPRM1; PRKCE; EGFR; PTGS1; HEXB; TYR; MME; BACE1; HPSE; PSEN2; CASP2; KCNH2; |
| 17 | Nucleoplasm | 6.201550388 | 2.011183084 | CDC25B; CSNK1A1; CDC25A; POLB; CDC45; PRKDC; RELA; CDK9; CDK2; MAPK1; CDK1; CDK4; CASP3; CASP6; CASP7; MAPK10; |
| 18 | Extracellular region | 6.201550388 | 2.043033679 | ACHE; MIF; SHBG; PTGFR; PLG; ALB; ERBB2; CDA; F2; MMP1; F10; SERPINE1; VEGFA; FGF1; FGF2; TLR9; |
| 19 | Centrosome | 5.426356589 | 1.204603067 | FASN; CDC25B; GSK3B; CSNK1A1; CSNK1D; G6PD; CDC45; PRKDC; GAPDH; MAPK1; HSPA8; CDK1; HSP90AA1; PSEN2; |
| 20 | Cell surface | 5.426356589 | 3.854600547 | KCNMA1; MIF; ABCB1; OPRM1; AOC3; CYP3A4; ADRA1A; ADA; STAT3; HSPA8; HSPA5; DPP4; ADAM17; VEGFA; |
| Molecular function | | | | |
| 1 | Catalytic activity | 17.76061776 | 6.051933686 | HMGCR; CA9; CA2; CA1; CA7; CA12; CA14; MAOB; FASN; MAOA; G6PD; AKR1B10; HSD11B1; HAO1; HSD17B3; GSR; CYP11B1; CYP11B2; CA3; CA6; CA13; CA5B; CYP2A6; NOX4; DAO; CYP3A4; PTGS1; CYP2D6; TK1; ADK; GAPDH; HK2; HK1; TYR; MANBA; GRK1; AKR1C1; CA4; CA5A; IDO1; AKR1C3; DTYMK; SQLE; ALPL; HSD11B2; HPSE; |
| 2 | G-protein coupled receptor activity | 10.42471042 | 2.568044778 | GABBR1; GPBAR1; DRD2; PTGER2; PTGFR; FFAR1; OPRM1; OPRK1; HTR2B; ADRA2A; ADRA2C; ADRA2B; DRD1; ADRA1D; HTR2A; HTR2C; DRD3; HTR6; ADRA1A; HTR1B; ADORA1; ADORA2A; ADORA3; ADORA2B; EDNRA; PTAFR; MLNR; |
| 3 | Protein serine/threonine kinase activity | 7.722007722 | 4.65187341 | ERN1; PIM3; GSK3B; CSNK1A1; CSNK1D; PRKDC; PRKCA; PRKCD; PRKCG; PRKCE; PRKCH; PRKCQ; CDK9; CSNK2A1; MAPK1; CDK1; PIM1; GRK6; MAPK10; PRKCB; |
| 4 | Transcription regulator activity | 5.019305019 | 1.094234676 | EP300; BRD2; HDAC3; HDAC2; HDAC1; HDAC5; HDAC4; HDAC9; NFKBIA; KDM4C; LGALS3; HDAC6; HDAC8; |
| 5 | Metallopeptidase activity | 4.633204633 | 8.320326316 | ADAMTS5; MMP2; MMP3; MMP9; ADAM17; MMP1; MME; ADAMTS4; MMP14; MMP13; MMP7; MMP8; |
| 6 | Transporter activity | 4.247104247 | 1.337577697 | MB; NPC1L1; FABP3; FABP5; FABP2; SERPINA6; SHBG; ALB; ABCB1; SLC5A1; AKR1C2; |
| 7 | Hydrolase activity | 3.861003861 | 3.450470901 | ACHE; CES1; AHCY; FUCA1; HEXA; HEXB; EPHX2; FBP1; MGAM; TREH; |
| 8 | Protein serine/threonine phosphatase activity | 2.702702703 | 11.14623955 | PPM1B; PPP1CA; PPP5C; PPP1CC; PPP2CA; PPP2R5A; PPM1A; |
| 9 | Oxidoreductase activity | 2.316602317 | 2.612061181 | CYP19A1; MPO; SRD5A1; NOS2; AKR1B1; AKR1C2; |
| 10 | Transcription factor activity | 1.930501931 | 0.416372655 | PPARD; VDR; RELA; STAT3; ESR1; |
| 11 | Auxiliary transport protein activity | 1.930501931 | 1.052791024 | SLC22A6; SLC5A2; SLC5A4; SLC29A1; SLC28A3; |
| 12 | Cysteine-type peptidase activity | 1.930501931 | 7.619863921 | CASP3; CASP6; CASP7; CASP8; CASP2; |
| 13 | DNA binding | 1.544401544 | 0.429063634 | PHF8; KDM5C; HDAC2; TLR9; |
| 14 | Chaperone activity | 1.544401544 | 2.226901891 | FABP4; MCL1; HSPA5; HSP90AA1; |
| 15 | Growth factor activity | 1.544401544 | 2.550785449 | TYMP; VEGFA; FGF1; FGF2; |
| 16 | Peptidase activity | 1.544401544 | 4.837302314 | PLG; F2; DPP4; F10; |
| 17 | Ligand-dependent nuclear receptor activity | 1.544401544 | 8.015193009 | AR; NR1H4; PPARA; RORC; |
| 18 | Protein tyrosine phosphatase activity | 1.544401544 | 11.2199883 | PTPN22; PTPN1; PTPN2; PTPN11; |
| 19 | Molecular function unknown | 1.158301158 | 0.037050748 | CISD1; LRRK2; PTGES; |
| 20 | Ubiquitin-specific protease activity | 1.158301158 | 0.558695726 | EGLN1; KDM2A; PSMB5; |

Table S3: the KEGG pathways of all targets of *Paeonia officinalis* the pathways are arranged in descending order according to enrichment FDR

| No. | Pathway | Enrichment FDR | no of Genes |
| --- | --- | --- | --- |
| 1 | Vascular smooth muscle contraction | 3.84E-11 | 16 |
| 2 | Proteoglycans in cancer | 2.67E-11 | 19 |
| 3 | Calcium signaling pathway | 7.65E-12 | 21 |
| 4 | CAMP signaling pathway | 1.34E-12 | 21 |
| 5 | Hepatitis B | 5.10E-13 | 19 |
| 6 | Measles | 3.46E-14 | 19 |
| 7 | Inflammatory mediator regulation of TRP channels | 1.90E-14 | 17 |
| 8 | Prostate cancer | 1.71E-14 | 17 |
| 9 | Nitrogen metabolism | 1.20E-14 | 10 |
| 10 | EGFR tyrosine kinase inhibitor resistance | 1.20E-14 | 16 |
| 11 | AGE-RAGE signaling pathway in diabetic complications | 1.87E-15 | 18 |
| 12 | Chemical carcinogenesis | 1.87E-15 | 23 |
| 13 | Lipid and atherosclerosis | 1.22E-15 | 24 |
| 14 | HIF-1 signaling pathway | 6.78E-16 | 19 |
| 15 | Insulin resistance | 6.60E-16 | 19 |
| 16 | Viral carcinogenesis | 5.67E-16 | 24 |
| 17 | Neuroactive ligand-receptor interaction | 5.00E-17 | 31 |
| 18 | MicroRNAs in cancer | 3.73E-18 | 24 |
| 19 | Pathways in cancer | 3.35E-24 | 45 |
| 20 | Metabolic pathways | 5.34E-30 | 80 |
